# Essential Domains of *Schizosaccharomyces pombe* Rad8 Required for DNA Damage Response

**DOI:** 10.1534/g3.114.011346

**Published:** 2014-05-28

**Authors:** Lin Ding, Susan L. Forsburg

**Affiliations:** Program in Molecular and Computational Biology, University of Southern California, Los Angeles, California 90089-2910

**Keywords:** SNF2 postreplication repair, helicases, ubiquitin E3 ligase, HIRAN domain

## Abstract

*Schizosaccharomyces pombe* Rad8 is a conserved protein homologous to *S. cerevisiae* Rad5 and human HLTF that is required for error-free postreplication repair by contributing to polyubiquitylation of PCNA. It has three conserved domains: an E3 ubiquitin ligase motif, a SNF2-family helicase domain, and a family-specific HIRAN domain. Data from humans and budding yeast suggest that helicase activity contributes to replication fork regression and template switching for fork restart. We constructed specific mutations in the three conserved domains and found that both the E3 ligase and HIRAN domains are required for proper response to DNA damage caused by a variety of agents. In contrast, mutations in the helicase domain show no phenotypes in a wild-type background. To determine whether Rad8 functionally overlaps with other helicases, we compared the phenotypes of single and double mutants with a panel of 23 nonessential helicase mutants, which we categorized into five phenotypic groups. Synthetic phenotypes with *rad8∆* were observed for mutants affecting recombination, and a *rad8* helicase mutation affected the HU response of a subset of recombination mutants. Our data suggest that the *S. pombe* Rad8 ubiquitin ligase activity is important for response to a variety of damaging agents, while the helicase domain plays only a minor role in modulating recombination-based fork restart during specific forms of replication stress.

Proper response to DNA damage during S phase requires that stalled replication forks are protected and efficiently restarted (Lambert and Carr 2013; Mirkin and Mirkin 2007). Evidence suggests that arrest and restart depend on management of single-stranded DNA and recombination. For example, on release from hydroxyurea (HU), which starves for nucleotides, *S. pombe* cells show a burst of foci of the single stranded DNA binding protein RPA, followed by foci of homologous recombination (HR) protein Rad52 (Carr and Lambert 2013; Meister *et al.* 2005; Sabatinos *et al.* 2012). This is consistent with HR involvement in fork restart (Bailis *et al.* 2008; Lambert *et al.* 2010; Meister *et al.* 2005). HR intermediates are processed and managed by specific helicases (*e.g.*, Rqh1; Fml1) and nucleases (*e.g.*, Mus81) to prevent inappropriate rearrangements (Dehe *et al.* 2013; Doe *et al.* 2002; Nandi and Whitby 2012; Sun *et al.* 2008; Willis and Rhind 2009).

There is good evidence from several systems that one form of fork restart works through a template switching pathway, with recovery through a Holliday junction-like structure (Atkinson and McGlynn 2009; Lambert and Carr 2013). Accumulation of RPA on ssDNA regulates this reaction (Betous *et al.* 2013; Sirbu *et al.* 2013). Several lines of evidence link the *S. cerevisiae* Rad5 to this activity. *Sc*Rad5 functions in the error-free branch of postreplication repair (PRR) by promoting the polyubiquitylation of PCNA (Branzei *et al.* 2004). It also has helicase-dependent fork reversal and restart activity, suggesting that it functions in fork regression downstream of PCNA ubiquitylation (Blastyak *et al.* 2007; Minca and Kowalski 2010). Cells with mutations that disrupt the ATP binding site in *ScRAD5* are sensitive to DNA damage and disrupt the ligation of broken ends in an MRN-dependent pathway (Chen *et al.* 2005; Minca and Kowalski 2010). *Scrad5* mutants are also HU-sensitive, consistent with a role in fork recovery (Kapitzky *et al.* 2010; Kats *et al.* 2009). Rad5 has two human homologs, SHPRH and HLTF (Figure 1B) (Unk *et al.* 2010). SHPRH promotes PCNA polyubiquitylation (Motegi *et al.* 2006b). HLTF facilitates template switching via its double-stranded DNA translocase activity (Blastyak *et al.* 2010). It also displaces RPA and PCNA from a modeled replication fork *in vitro* and contributes to Rad51-independent D-loop formation (Achar *et al.* 2011; Burkovics *et al.* 2014). SHPRH and HLTF respond to different forms of damage (Lin *et al.* 2011). Together, these data suggest that the Rad5/HLTF helicase domain contributes to replication fork stability and restart, possibly in an alternative pathway to Rad51.

**Figure 1:**
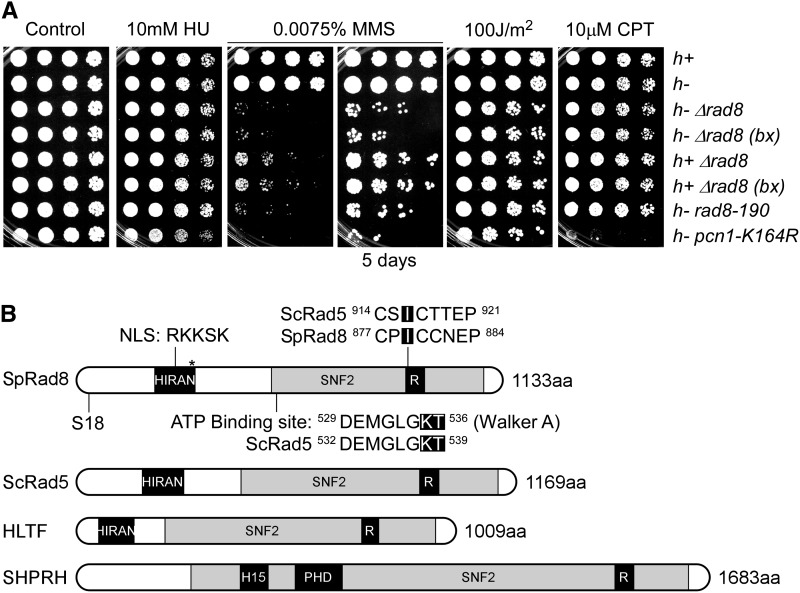
Rad8 is required for response to MMS-induced damage. (A) Representative response to MMS assessed by serial dilution. Strains were grown overnight at 32°, 1:5 serially diluted, and spotted to plain YES rich medium (Control) and YES with indicated drugs. Plates were incubated at 32° for 3 d unless otherwise indicated. bx = FY5627 was backcrossed once to FY528 (wild-type). These assays were repeated five times. (B) Schematic representation of Rad8 functional domains. S18 is a potential phosphorylation site. *Represents the protein product of *rad8-190*, which has a premature stop codon at amino acid 315. R, RING finger domain; H15, linker histone 1 and histone 5 domains; PHD, PHD-finger.

In *S. pombe*, the orthologous protein Rad8 (Figure 1B) has been shown to extend the ubiquitin chain on mono-ubiquitinated PCNA on K164 (Frampton *et al.* 2006), but the role of its ATP-dependent helicase domain has not been assessed. We performed a structure-function analysis of *Sp*Rad8. Surprisingly, and in contrast to data from budding yeast, we found no evidence for a role of the Rad8 ATPase domain in the response to replication stress in *S. pombe* in otherwise wild-type cells. Instead, the ubiquitin ligase domain is required for Rad8 to promote genome stability in response to a variety of stresses, suggesting that PCNA modification is required for multiple facets of genome maintenance. We used a candidate approach to investigate whether the putative Rad8 helicase overlaps with other helicase genes in *S. pombe*. An analysis of the drug sensitivity of a panel of nonessential helicase mutants allows identification of five distinct phenotypic groups, suggesting specialized helicase functions for specific types of replication stress. There are synthetic interactions between *rad8∆* and a subset of genes involved in homologous recombination repair and recombination-mediated fork restart. The only phenotype associated with the helicase domain of Rad8 is in HU, and only in the absence of certain HR activities. This implicates PCNA modification in a wide range of damage response pathways, and suggests that fork regression or D-loop formation mediated by Rad8 helicase is only a minor player in replication fork restart in fission yeast.

## Materials and Methods

### Strain construction

All *S. pombe* strains were constructed and maintained in yeast extract plus supplement (YES) medium or under selection in Edinburgh minimal media (EMM) with appropriate supplements using standard techniques (Sabatinos and Forsburg 2010).

*S. pombe* strains (listed in Supporting Information, Table S1) were from our collection or purchased from the Bioneer Corporation and the Korea Research Institute of Biotechnology and Bioscience. The *∆rad8*::*kanMX-Bioneer* (FY5132) deletion was isolated from the Bioneer *S. pombe* Deletion Mutant Library (V2-11-F11). It was backcrossed twice with laboratory wild-type strains. Both mating types were retained (FY5216: *h^+^* or FY5217: *h^−^*). *∆rad8*::*hphMX* (FY5625: *h^+^* or FY5627: *h^−^*) was generated by replacing the kanamycin-resistant fragment of the Bioneer deletion with hygromycin B–resistant marker (Hentges *et al.* 2005). *∆rad8*::*hphMX* or backcrossed *∆rad8*::*kanMX-Bioneer* was used to construct double mutants. Strains were generated by tetrad dissection or random spore analysis.

Genomic DNA was extracted using a LioAc-SDS–based method that was derived and optimized from (Looke *et al.* 2011) for *S. pombe*. Briefly, in a 1.5-mL eppendorf tube, a single colony was resuspended in 100 μL LiOAc-SDS buffer (200 mM LiOAc, 1% SDS) and incubated at 70° for 15 min. Three volumes (300 μL) of 96% ethanol were added to the sample. After vortexing, the mixture was centrifuged at 15,000*g* for 3 min. The pellet was completely air-dried, resuspended in 100 μL TE, and centrifuged at 15,000*g* for 3 min. One microliter of the supernatant was used as template in a 20-μL colony PCR reaction.

### Cloning of *rad8^+^* gene and plasmid construction

The *rad8^+^* gene was cloned in two steps. First, two independent fragments, one from ATG to a naturally occurring *Spe*I site within the ORF (*rad8A*) and the other from the *Spe*I site to the TAA codon (*rad8B*), were amplified by PCR and cloned into a pBlueScript vector. The *Xho*I site in the *rad8B* was silenced by site-directed mutagenesis. The complete *rad8^+^* ORF was generated by ligating the *rad8A* fragment into *rad8B-noXhoI* containing plasmid at *Xho*I and *Spe*I. Mutations were introduced separately in *rad8A* or *rad8B-noXhoI* containing plasmids prior to generation of the full gene. Phusion Site-Directed Mutagenesis Kit (Finnzymes) was used for site-directed mutagenesis. Plasmids are listed in Table S2.

### Constructing *rad8* mutants at its endogenous locus

The *rad8* mutants were created as described (Watson *et al.* 2008). The base strain (FY5622: *h^−^ Δrad8*::*loxP-ura4^+^-loxM3 ura4-D18 leu1-32 ade6-M210 can1-1*) was constructed by replacing the entire *rad8^+^* ORF (from start to stop codon) with the *loxP-ura4^+^-loxM3* cassette that was amplified from the pAW1 plasmid (EUROSCARF-P30537) using primers *TATACATGTTATTTTATATTTCTACAGTTTTTGGTAGCTTAAAGTTTGGATAAGCAAACATTACCAAGAAACTCAATAAA****CGGATCCCCGGGTTAATTAA*** and *GTAGCAATTGCATTTCATATGCATAATATGAAAATACTTTTTTTTTACGATAGCTTTTAATCGGCTTGGTGAAACCGTTG****GAATTCGAGCTCGTTTAAAC***. Bold sequences indicate the sequences homologous to pAW1. The wild-type (pLD35) and mutated *rad8* coding plasmids (pLD36, pLD37, pLD38, and pLD39) were digested with *Xho*I and *Sac*I and cloned into the pAW8-*Xho*I plasmid (EUROSCARF-P30585). The resulting plasmids [pLD45: *rad8^+^*; pLD46: *rad8-∆HIRAN*; pLD47: *rad8-K535A*, *T536A* (*rad8-HD*); pLD48: *rad8-I879A* (*rad8-LD*); and pLD49: *rad8-K535AT536AI879A* (*rad8-HDLD*)] were transformed into the base strain and selected on EMM+Ade+thiamine plates. Transformants were grown in nonselective thiamine-free medium at 32° for 1 d, plated onto 5′-FOA plates, and incubated at 32° for 4 d. The 5-FOA–resistant and LEU*^−^* candidates were confirmed by colony PCR and verified by sequencing.

### Serial dilution assays

Cells were grown to mid-log phase in YES. Five-fold serial dilutions were prepared in YES and spotted on drug containing rich medium. The plates were incubated 5 d at 25° or 3 d at 32°. Experiments were repeated two to five times.

### Protein extracts and immunoblotting

Whole cell extracts (WCE) were prepared using trichloroacetic acid (TCA) extraction as described (Catlett and Forsburg 2003). Eighty micrograms of WCE were separated by SDS-PAGE in a 6% acrylamide gel. FLAG tag was detected with mouse anti-FLAG M2 (1:1000; Sigma) and anti-mouse-IgG-HRP (1:2000; Sigma). Mcm7 was detected with antibody purified from rabbit serum 6184 (1:1000) (Liang and Forsburg 2001) and anti-rabbit-HRP (1:2000; BD Biosciences).

### GFP fusions

Two types of GFP fusion were made. For Western blot, the C-terminus of *rad8^+^* was tagged with a *GFP* fragment at its native locus as described (Bahler *et al.* 1998). For microscopy (overexpression), linearized plasmids that carry *rad8-GFP* (under the *nmt1* promoter), either wild-type or mutant derivatives, were integrated at *leu1-32* locus in *rad8∆* background. The *nmt1* promoter was repressed in the presence of 15 μM thiamine (Maundrell 1993).

### Microscopy

GFP tagged Rad8 strains were grown overnight in EMM-LEU containing 15 μM thiamine. Cells were harvested, washed twice with EMM-LEU, and released in thiamine-free medium to allow overexpression. Pictures were taken 16 hr after induction with a DeltaVision Core epifluorescence wide-field microscope (Applied Precision, WA) using GFP/mCherry Chroma ET C125705 filter (Ex 520/50; Em 630/80; polychroic mirror) and oil-immersion Olympus 60× lens (1.4 NA). Fifteen pictures of z-sections at 0.3 μm were captured, deconvolved, and then projected in softWoRx 5.5. The final figures were cropped and assembled in Canvas 12 (ACD Systems).

## Results

### Rad8 is required for response to a subset of genome damaging agents

To assess the role of Rad8 in damage response, we examined *rad8∆* sensitivity to different genotoxins (Figure 1A) and compared this to a commonly used *rad8* truncation allele, *rad8-190*, which we determined encodes a protein product with a premature stop codon at amino acid 315. We compared these to a nonubiquitinable mutant of *pcn1* (*pcn1-K164R*) (Frampton *et al.* 2006). *rad8∆* has no growth defect in the absence of drugs, and no evidence of a meiotic defect (data not shown). As seen previously, it is very sensitive to MMS and slightly sensitive to UV (Doe *et al.* 1993). *rad8∆* MMS sensitivity is not as severe as *pcn1-K164R*, probably because the error-prone pathway that depends on single ubiquitylation of PCNA is still functional in *rad8∆* (Frampton *et al.* 2006). Surprisingly, and in contrast to *S. cerevisiae ScRad5*, *rad8∆* mutants are not sensitive to HU. We found that *rad8∆* is also not sensitive to CPT, a topoisomerase toxin that causes S phase-specific DNA breaks (Wan *et al.* 1999). However, *pcn1-K164R* is very CPT-sensitive, possibly due to a sumoylation-mediated pathway involving the same residue (Kai *et al.* 2007).

Interestingly, *h^−^* ∆*rad8* has increased sensitivity compared with *h^+^* ∆*rad8* when exposed to MMS (Figure 1A). Mutants lacking homologous recombination proteins often are sicker in *h^−^* than in *h^+^* configuration, which is presumed to reflect the absence of a template for repair of the mating type imprint and break required for switching in the *h*^−^ strain (Khasanov *et al.* 1999). This could suggest a role for Rad8 in aspects of HR repair. We compared *h^−^* strains throughout the remainder of this study to avoid differences attributable to mating type.

### Domain structure of Rad8

*S. pombe* Rad8 was first identified as a member of the SNF2 helicase family based on sequence homology (Doe *et al.* 1993) and has distinct domains matching those in ScRad5 and HLTF (Minca and Kowalski 2010; Unk *et al.* 2010). There is a SNF2 helicase domain including an ATP binding site. Mutations that change the lysine and threonine to alanine (K538A, T539A) in the ATP binding site abolish the helicase activity in *Sc*Rad5 (Chen *et al.* 2005; Minca and Kowalski 2010). Embedded in the helicase domain, there is a RING-type Zinc finger ubiquitin E3 ligase domain that polyubiquitinates Pcn1 (ScPol30, hPCNA) in concert with the Mms2/Ubc13 E2 heterodimer (Frampton *et al.* 2006). A point mutation from isoleucine to alanine (I916A) in this motif in *Sc*Rad5 abolished the E3 ubiquitin ligase activity by eliminating its interaction with Ubc13 (Ulrich 2003). Near the N-terminus is an uncharacterized HIRAN domain, a motif shared by all the members of this family (HIP116, Rad5p N-terminal domain). It has been suggested that this domain recognizes specific DNA damage or stalled replication forks (Iyer *et al.* 2006), but it has not been analyzed. The Rad8 HIRAN domain spans amino acid 206 to 319 and contains a potential NLS (nuclear localization signal) RKKSK between amino acids 245 and 251. The *rad8-190* truncation allele expresses most of this domain (amino acid 1-314) while deleting the helicase and RING-finger E3 ligase domains. We constructed mutations (Figure 1B) in all three of these conserved domains to assess their function in *S. pombe*. A large-scale fission yeast phosphorylation analysis (Wilson-Grady *et al.* 2008) suggested that a serine residue (S18) of Rad8 is a potential phosphorylation site. However, we observed no phenotype of an S18A mutation in DNA damage response, so this was not investigated further (Figure S1).

### The HIRAN domain contributes to nuclear localization

**Figure 2:**
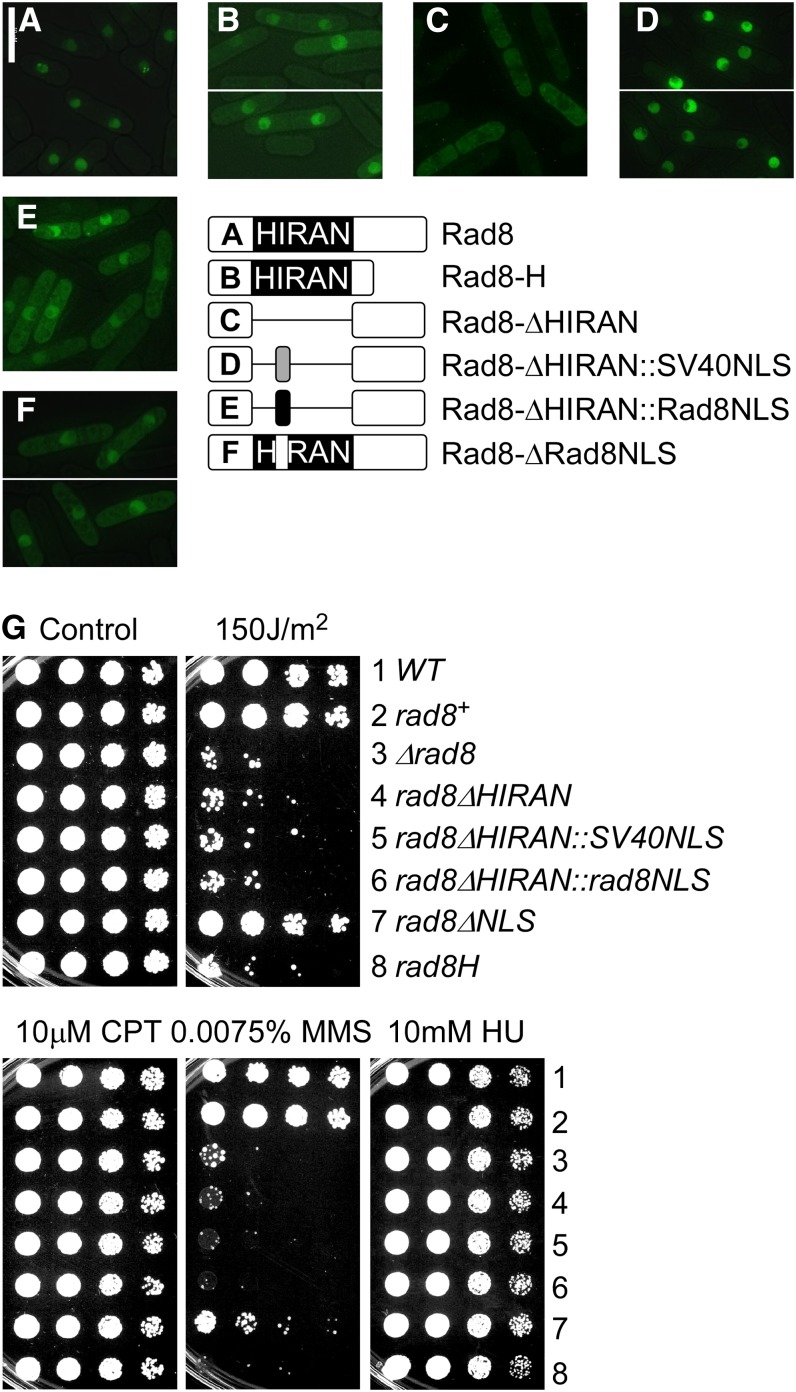
Nuclear localization is necessary but not sufficient for Rad8 function. (A–F) Schematic representation and localization of Rad8-GFP proteins, overproduced from a single copy transgene integrated at *leu1-32* in *∆rad8* background. Pictures were taken 16 hr after the removal of thiamine: (A) Rad8-GFP; (B) Rad8-HIRAN-GFP; (C) Rad8-∆HIRAN; (D) Rad8-∆HIRAN::SV40NLS; (E) Rad8-∆HIRAN::rad8NLS; and (F) Rad8-∆NLS (with schematic representations of Rad8 HIRAN–related constructs on the right; not drawn to scale). (G) Drug sensitivity of indicated mutants. These mutants were integrated without a GFP tag at the native locus under the endogenous promoter. Strains were grown overnight at 32°, 1:5 serially diluted, and spotted to plain YES rich medium (Control) and YES with indicated drugs. (1) Wild-type. (2) *loxP-rad8^+^-loxM3*. (3) *loxP-∆rad8-loxM3*. (4) *loxP-rad8-∆HIRAN-loxM3*. (5) *loxP-rad8∆HIRAN*::*SV40NLS-loxM3*. (6) *loxP-rad8∆HIRAN*::*rad8NLS-loxM3*. (7) *loxP-rad8-∆NLS-loxM3*. (8) *loxP-rad8-HIRAN-loxM3*. Plates were incubated at 32° for 3 d.

To assess protein location, we tagged endogenous Rad8 with GFP at its C-terminus. Under the endogenous promoter, there was not sufficient signal to visualize the protein (data not shown). Therefore, we engineered overproduction strains to increase the GFP signal. We integrated Rad8-GFP at the at *leu1^+^* locus, under the control of the *nmt1*^+^ promoter. This promoter allows modest levels of expression in thiamine, and a dramatic overproduction in the absence of thiamine (Forsburg 1993). We observed a distinctive Rad8-GFP signal in the nucleus within 16 hr of removing thiamine (Figure 2A). Prolonged Rad8-GFP overproduction over several days is slightly toxic (Figure S2A).

We constructed a 3′ truncation allele *rad8-H* mutant that expresses only the N-terminus of Rad8 up to the end of the HIRAN domain, and a HIRAN deletion mutant (*rad8-∆HIRAN*) that precisely deletes aa206-319. Rad8-H-GFP (integrated into the *leu1^+^* locus under the *nmt1* promoter in the *rad8∆* strain) localizes in the nucleus (Figure 2B), whereas Rad8-∆HIRAN-GFP remains in the cytoplasm (Figure 2C). Next, we replaced the entire HIRAN domain with the SV40NLS (**PKKKRKV**) (Pasion and Forsburg 1999), and this restored the nuclear localization of Rad8 (Figure 2D). However, inserting the putative Rad8NLS (RKKSK) (Doe *et al.* 1993) in the same configuration only partially restored the nuclear localization (Figure 2E). A smaller deletion (amino acids 246 to 250) removed the RKKSK sequence (Rad8-∆NLS). The majority of this protein localized properly, indicating this is not the primary NLS (Figure 2F).

To test the drug sensitivity of the HIRAN mutants, we integrated them without GFP under the endogenous *rad8^+^* promoter at the native locus using the Cre recombinase–mediated cassette exchange (RMCE) system (see *Materials and Methods*) (Watson *et al.* 2008). Both *rad8-H* and *rad8-∆HIRAN* phenocopy *rad8∆* in damage sensitivity (Figure 2G). Importantly, restoration of nuclear localization with SV40NLS did not rescue the drug sensitivity. The Rad8-∆NLS strain was modestly sensitive to damage, consistent with the slight defect in localization (Figure 2G). Taken together, these results suggest that nuclear localization is necessary but not sufficient for Rad8 function, and that the HIRAN domain provides additional functions beyond nuclear localization.

### Mutations in the E3 ligase and helicase domains have different phenotypes

**Figure 3:**
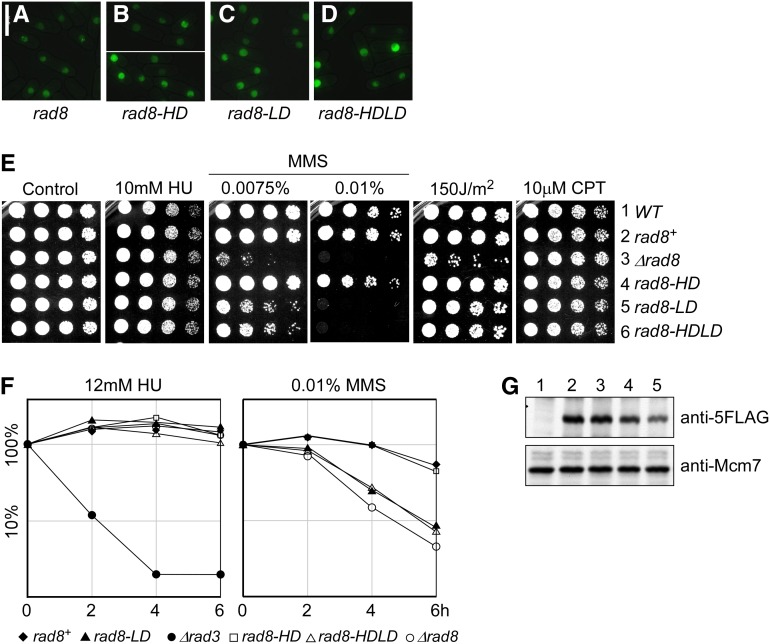
Ring finger is essential for Rad8 damage response. (A–D) Localization of Rad8-GFP proteins, overproduced from a single copy transgene integrated at *leu1-32* in *∆rad8* background. Pictures were taken 16 hr after the removal of thiamine: (A) Rad8-GFP; (B) Rad8-HD-GFP; helicase dead; (C) Rad8-LD-GFP, ubiquitin ligase dead; and (D) Rad8-HDLD-GFP, double mutant. (E) Drug sensitivity of indicated mutants. These mutants were integrated without a GFP tag at the native locus under the endogenous promoter. Strains were grown overnight at 32°, 1:5 serially diluted, and spotted to plain YES rich medium (Control) and YES with indicated drugs. (1) Wild-type. (2) *loxP-rad8^+^-loxM3*. (3) *loxP-∆rad8-loxM3*. (4) *loxP-rad8-K535AT536A-loxM3*. (5) *loxP-rad8-I879A-loxM3*. (6) *loxP-rad8-K535AT536AI879A-loxM3*. Plates were incubated at 32° for 3 d. (F) Representative relative survival curves of indicated mutants to acute drug exposure. HU (left), MMS (right). *rad3Δ* MMS survival curve was not plotted due to complete loss of viability at the 2-, 4-, and 6-hr time points. For each condition, two biological repeats were conducted. (G) Protein level of *rad8* mutants. *rad8* mutants were tagged with 5FLAG C terminally. Whole cell lysates were prepared using TCA extraction. Mcm7 was used as a loading control. Lane 1: *wild-type*; lane 2: *rad8-5FLAG*; lane 3: *loxP-rad8-5FLAG*; lane 4: *loxP-rad8-HD-5FLAG*; and lane 5: *loxP-rad8-LD-5FLAG*.

Using the same strategies, we examined localization and drug sensitivity of Rad8 proteins containing point mutations in the putative helicase or ring finger domains. The point mutants correspond to the separation-of-function mutants in *S. cerevisiae* (Chen *et al.* 2005; Minca and Kowalski 2010). Similar to *rad8^+^*, the helicase-dead mutant *rad8-K535A*, *T536A* (*rad8-HD*), ubiquitin ligase-dead mutant *rad8-I879A* (*rad8-LD*), and the mutant with all three mutations *rad8-K535AT536AI879A* (*rad8-HDLD*) were all nuclear localized (Figure 3, A–D). The *Pnmt1-rad8-GFP* fully complemented *∆rad8* and cells mounted the same response to damaging drugs in the presence of thiamine (Figure S2B). However, both wild-type and mutant forms of Rad8 show a slight reduction in growth and increased sensitivity to drugs when strongly overproduced in thiamine-free medium (Figure S2C). This could be due to toxicity or a media effect. Interestingly, because we see a similar effect with both HD and LD mutants, this does not appear to be related to catalytic activity but could reflect a function for the HIRAN domain or a structural role.

We integrated the mutants at the endogenous locus without the GFP tag and under the natural promoter. Surprisingly, and different from *S. cerevisiae* (Minca and Kowalski 2010), *rad8-HD* mutant did not affect growth in chronic or acute treatment with MMS (Figure 3 E and F). The Rad8 ubiquitin ligase domain proved to be the major contributor to all damage responses (Figure 3 E and F). The amount of Rad8 protein in these mutants is similar (Figure 3G).

*Sc*Rad5 channels the PRR to the error-free sub-pathway by polyubiquitinating Pcn1 (ScPol30/PCNA) and promoting fork reversal (Blastyak *et al.* 2007; Ramasubramanyan *et al.* 2010; Unk *et al.* 2010). Previous work with *S. pombe* showed that SpRad8 is required for PCNA polyubiquitylation (Frampton *et al.* 2006). We compared the phenotypes of *rad8∆* and the *rad8* point mutations in combination with different PRR mutants and examined their sensitivity to MMS, HU, and UV. As expected, *rad8∆* is in a common epistasis group with *rhp18∆* and *mms2∆* (Figure S3A), whereas it has increased sensitivity to MMS when combined with mutations in the TLS polymerases in the error-prone arm of the pathway (Figure S3B).

**Figure 4:**
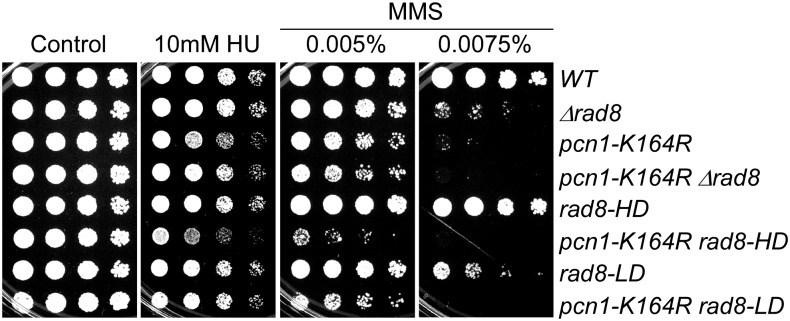
A mutation in Rad8 ligase domain responsible for genetic interactions with *pcn1-K164R*. Strains were grown overnight at 32°, 1:5 serially diluted, and spotted to plain YES rich medium (Control) and YES with indicated drugs. Plates were incubated at 32° for 3 d unless otherwise indicated.

As seen previously (Frampton *et al.* 2006), *pcn1-K164R* (which cannot be ubiquitylated) is epistatic to *rad8∆*. We observed a similar phenotype for *rad8-LD*, which lacks the ubiquitin ligase domain (Figure 4). Although we observe no obvious phenotype of the helicase mutant alone, the *pcn1-K164R rad8-HD* double mutant has reduced viability in response to MMS compared with *pcn1-K164R rad8∆* (Figure 4). Moreover, *pcn1-K164R rad8-HD* is slightly sensitive to HU. This suggests that the helicase domain may become important in response to replication stress only if PCNA cannot be modified at K164.

### A damage fingerprint of helicase mutants

**Figure 5:**
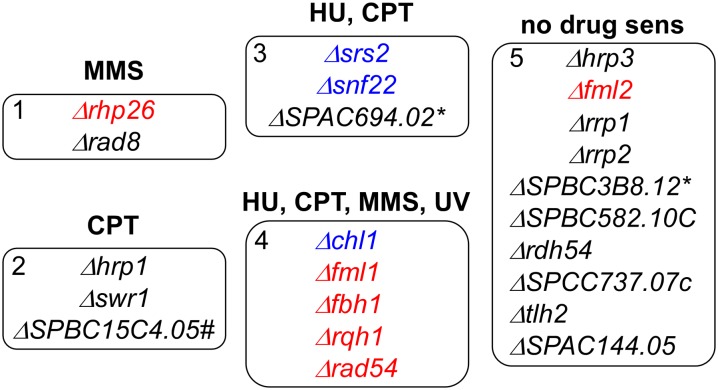
Nonessential helicases cluster in five phenotypic groups. Based on sensitivity to different genotoxins as seen in the serial dilution experiments (see Figure 6, Figure 7, Figure S3, and Figure S4 for data). Red font indicates strains that, when combined with *Δrad8*, show increased sensitivity to all damaging agents. Blue font indicates strains that, when combined with *Δrad8*, show mixed phenotypes of decreased and increased sensitivity to damaging agents. Black font indicates strains combined with *Δrad8* show no sensitivity to damaging agents. SPBC3B8.12 = SPBC11C11.11C. #RNA/DNA helicase. *RNA helicase.

**Figure 6:**
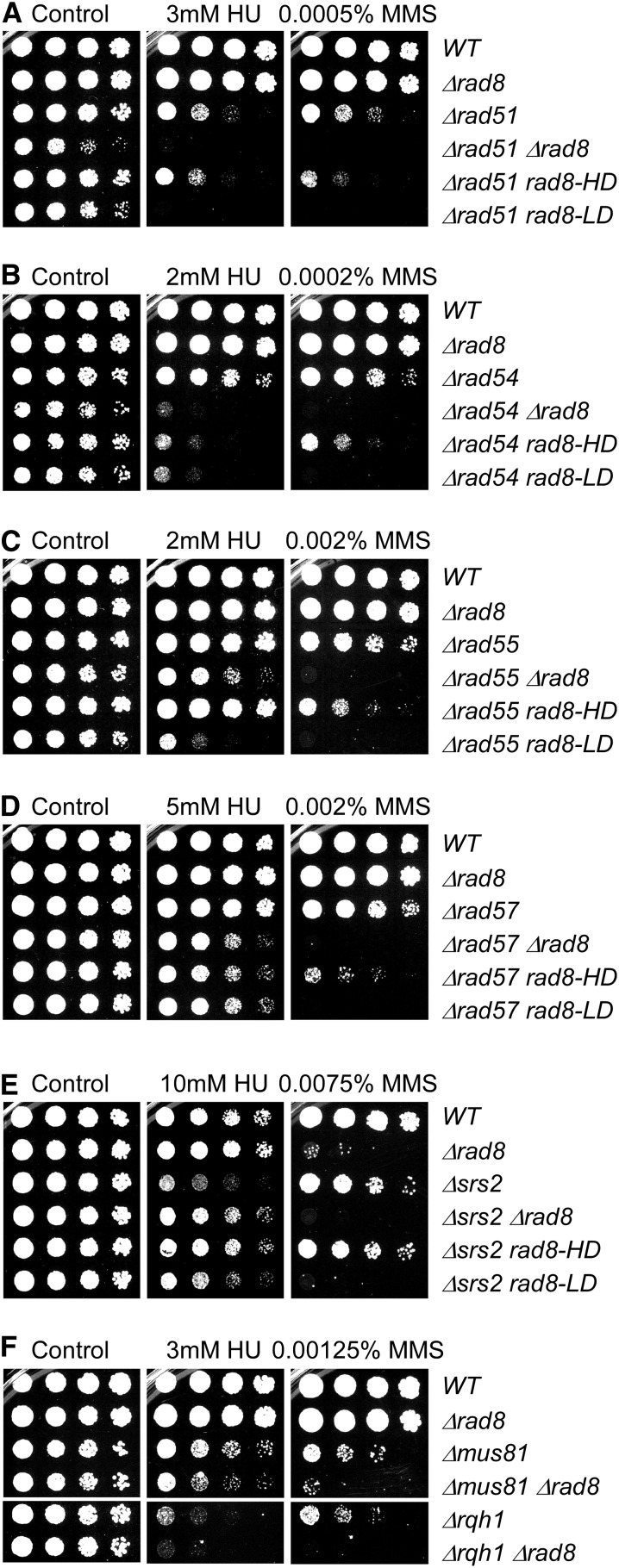
*rad8* genetically interacts with genes involved in homologous recombination. Strains were grown overnight at 32°, 1:5 serially diluted, and spotted to plain YES rich medium (Control) and YES with indicated drugs. Plates were incubated at 32° for 3 d.

**Figure 7:**
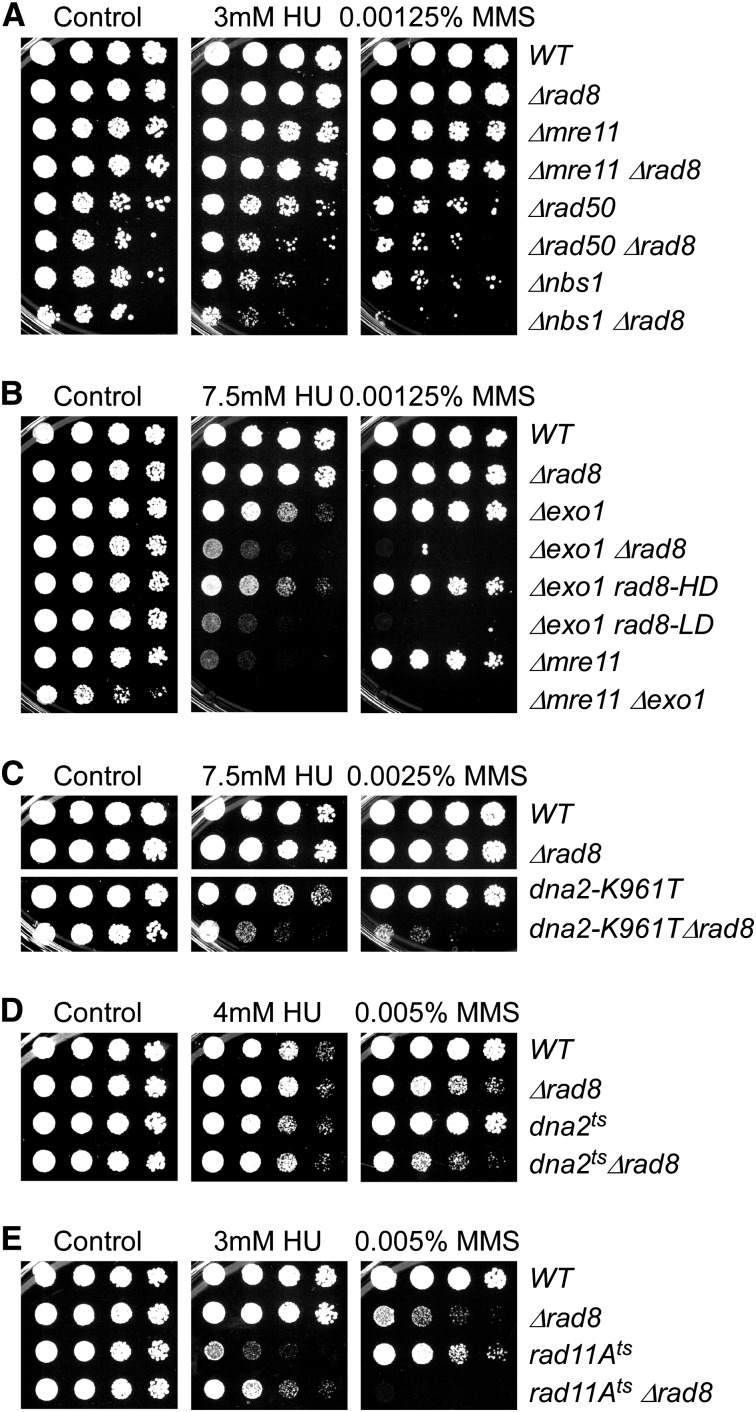
Rad8 ubiquitin ligase may contribute to resection. (A–C) Strains were grown overnight at 32°, 1:5 serially diluted, and spotted to plain YES rich medium (Control) and YES with indicated drugs. Plates were incubated at 32° for 3 d. (D) and (E) Strains were grown overnight at 25°, 1:5 serially diluted, and spotted to plain YES rich medium (Control) and YES with indicated drugs. Plates were incubated at 25° for 5 d.

Given the observations with the budding yeast and human orthologs, we were surprised at the absence of any phenotypes associated with the *rad8-HD* mutant. We investigated whether Rad8 is redundant with other helicases in *S. pombe*, which has 23 annotated nonessential helicases. First, we isolated mutants available in the Bioneer deletion collection (Deshpande *et al.* 2009) and compared their responses to DNA-damaging drugs or treatment. Based on their patterns of sensitivity to different DNA damaging agents, we identified five distinct groups (summarized in Figure 5; see references in Table S3 and data in Figure 6, Figure 7, Figure S4, and Figure S5).

The first group is specifically sensitive to MMS, consisting only of *rad8∆* itself and *rhp26∆*, the ortholog of *Sc*Rad26 associated with transcription-coupled repair (Kanamitsu and Ikeda 2011). Group 2 mutants were only sensitive to CPT treatment, but not the other agents. This group included two chromatin remodelers and an uncharacterized RNA/DNA helicase. Group 3 contained *snf22∆*, another member of the SNF2 family, *srs2∆*, and an uncharacterized putative RNA helicase *∆SPAC694.02*. They were sensitive to HU and CPT, which are specific to stress during S phase. The group 4 strains, sensitive to all agents tested, have been linked to aspects of replication fork stabilization and recombination. The last group of mutants showed no sensitivity to any of the agents we tested.

We examined the sensitivity of these mutations in combination with *rad8∆* (summarized in Figure 5; see references in Table S4 and data in Figure 6, Figure 7, Figure S4, and Figure S5). Not surprisingly, the *rhp26∆ rad8∆* double mutant was profoundly sensitive to MMS. There were no significant synthetic phenotypes observed in combination with group 2 mutants or the group 3 mutant *SPAC694.02∆*. Synthetic phenotypes were observed with most of the group 4 strains, generally showing increased sensitivity to all damaging agents (indicated in red). In a few cases, the double mutant improved growth on one agent (shown in blue): *srs2∆ rad8∆* and *snf22∆ rad8∆* show improved growth on HU (Figure 6E and Figure S4), whereas *fml1∆ fml2∆ rad8∆* (Figure S5) and *chl1∆ rad8∆* (Figure S4) both show improve growth on CPT.

We also examined conditional alleles of several essential helicases, including *dna2* (discussed below) and *pfh1*, which is required to replicate though particular structures in the genome (Pinter *et al.* 2008; Sabouri *et al.* 2012; Steinacher *et al.* 2012; Tanaka *et al.* 2002). *pfh1-R20* is a cold-sensitive allele (Boule and Zakian 2006). The *pfh1-R20 rad8∆* mutant showed increased sensitivity to MMS and UV, no effect on CPT, and a partial rescue on HU (Table S4 and Figure S4).

Based on the results from this analysis, we took a candidate approach to investigate whether the *rad8* helicase domain is responsible for any of the synthetic phenotypes we identified.

### Identifying an effect of the Rad8-helicase mutant in recombination-deficient backgrounds

Homologous recombination is a central component of the response to replication stress (Bailis *et al.* 2008; Lambert *et al.* 2010; Meister *et al.* 2005). *Sc*Rad5 and human HLTF are implicated in formation of recombination structures between sister chromatids that can promote replication fork restart, independent of Rad51 (Burkovics *et al.* 2014; Hu *et al.* 2013; Minca and Kowalski 2010; Mott and Symington 2011). We examined mutations affecting Rad51, which coats an invading ssDNA filament (Heyer *et al.* 2010). Filament formation is promoted by mediator complexes including Rad55/Rad57 and Sfr1/Swi5, and is opposed by the helicase Srs2 (Haruta *et al.* 2008; Heyer *et al.* 2010)). Invasion and D loop formation are promoted by recruitment of the Rad54 helicase (Heyer *et al.* 2010).

Consistent with previous observations (Frampton *et al.* 2006), we find that *rad51∆ rad8∆* is growth-impaired even without external genotoxins (Figure 6A). This growth defect was not as dramatic in the *rad51∆ rad8-LD* double mutant lacking the ubiquitin ligase domain and not apparent at all in the *rad51∆ rad8-HD* double mutant lacking the helicase motif, thus implicating the E3 ligase domain as an important contributor to genome stability in the absence of Rad51.

Both *rad51∆ rad8∆* and in *rad51∆ rad8-LD* show a dramatic increase in MMS sensitivity, which is consistent with loss of two pathways of repair (template switching and recombination). Increased HU sensitivity was also observed for both *rad51∆ rad8∆* and *rad51∆ rad8-LD* compared with *rad51∆* (note the relatively low dose used, because *rad51∆* is extremely HU-sensitive). Although the effect was modest, we observed a slight increase in MMS sensitivity in the *rad51∆ rad8-HD* double mutant. However, there was no synthetic phenotype on HU of *rad51∆ rad8-HD*.

We examined other members of the HR pathway: *rad54∆*, *rad55∆*, and *rad57∆*. On MMS, we saw similar phenotypes to those for *rad51∆*: a significantly increased sensitivity in double mutants with *rad8∆* or *rad8-LD* lacking the ligase domain and a very slightly increased sensitivity with the *rad8-HD* mutation in the helicase domain (Figure 6, B–D). This suggests that they all act in a common pathway with Rad51 for MMS response, and this implicates the helicase domain for a minor contribution in the MMS response when the other pathways are compromised.

However, phenotypes of these additional *rad* mutants were all different on HU. First, *rad54∆* is not quite as HU-sensitive as *rad51∆*, whereas *rad55∆* and *rad57*∆ are not sensitive at all (Figure S6). Second, there was a strikingly increased HU sensitivity of *rad54∆* in all the *rad8* double mutants, suggesting that both ligase and helicase activity are important in the absence of Rad54. In contrast, *rad55∆* showed increased HU sensitivity combined with *rad8∆* or especially *rad8-LD*, but no effect of *rad8-HD*, whereas *rad57∆* showed only a slight increase in sensitivity to the same extent in all the *rad8* double mutants. These data suggest that Rad8 helicase activity may be specifically important for the response in HU if Rad54 is missing, and suggest separable roles of the Rad55/57 mediator components.

Srs2 helicase is an anti-recombinase that opposes Rad51 filament formation (Macris and Sung 2005). However, Srs2 also promotes fork reversal in repetitive sequences (Kerrest *et al.* 2009) and contributes to fork restart and template switching at stalled forks (Lambert *et al.* 2010). In MMS, Srs2 is implicated in restraint of the HR response to promote PRR in budding yeast, but not in fission yeast (Doe and Whitby 2004; Kai *et al.* 2007). We observed that *rad8∆*, *srs2∆ rad8∆*, and *srs2∆ rad8-LD* all showed similar sensitivity to MMS and UV, whereas *srs2∆ rad8-HD* resembled the more modest phenotype of *srs2∆* (Figure 6E and Figure S4). Intriguingly, loss of *rad8* partly suppressed the HU sensitivity observed in *srs2∆*, and this suppression was strongest in the *srs2∆ rad8-HD* double mutant lacking the helicase. This is opposite the phenotype observed for *rad51∆ rad8*, *rad54∆ rad8*, and *rad55∆ rad8* double mutants, and suggests that the Rad8 helicase may antagonize Srs2.

Based on this result, we examined two additional proteins implicated in fork restart and HU response: the Mus81 resolvase and the Rqh1 helicase (Doe *et al.* 2002; Roseaulin *et al.* 2008). The double mutants with *rad8∆* showed increased defects in growth on HU, MMS, and UV (Figure 6F and Figure S4).

### Fml1 operates in an independent pathway

Another interacting helicase revealed in our screen is Fml1 (ScMph1/FANCM). This protein family is capable of fork reversal and promotes recombination at stalled replication forks (Blackford *et al.* 2012; Gari *et al.* 2008; Nandi and Whitby 2012; Prakash *et al.* 2005; Schurer *et al.* 2004; Sun *et al.* 2008; Zheng *et al.* 2011). Recent work suggests that budding yeast Mph1/FancM operates downstream of ScRad5 in repair of interstrand crosslinks (Daee *et al.* 2012). *S. pombe* has two Fml proteins: Fml1 and its paralogue, Fml2, which plays a minor role (Sun *et al.* 2008).

We examined the drug sensitivity of double and triple mutants (Figure S5). These showed no obvious growth defect on plate assays in the absence of replication stress, although the triple mutant was slightly elongated and grew more slowly in liquid media (data not shown). *fml1∆ rad8∆* shows an increased sensitivity to MMS, UV, CPT, and HU, relative to both parents. The triple mutant *fml1∆ fml2∆ rad8∆* is hypersensitive to MMS, UV, and HU, indicating a role for Rad8 in HU response when the Fml proteins are missing. *pcn1-K164R fml1∆ fml2∆* phenocopies *fml1∆ fml2∆ rad8∆*. The *rad8-HD* mutant rescues the drug sensitivity of the *fml1∆ fml2∆ rad8∆* triple mutant, and this suppression is abolished by *pcn1-K164R*, indicating that it is dependent on PCNA modification. Similar results were observed in HU, suggesting that the drug-sensitive phenotype of *rad8∆ fml∆* mutants in all these cases is linked to ubiquitylation of PCNA.

### Genetic evidence that Rad8 contributes to resection

Homologous recombination initiates from DNA ends, and broken ends are a target for resection by the MRN complex. There is evidence that *Sc*Rad5 binds to ssDNA as part of an end-joining pathway that involves the MRN complex (Chen *et al.* 2005). In contrast to the phenotype of Rad51 pathway members, we found only slight increase in MMS or UV sensitivity in double mutants between *rad8∆* and some MRN components (*rad50∆* and *nbs1∆*), suggesting a possible common function (Figure 7 and Figure S4).

MRN and the associated Ctp1 protein promote short-range resection, whereas long-range resection is promoted by Exo1 (Langerak *et al.* 2011). We observed that *exo1∆ rad8∆* double mutants have a substantial increase in MMS sensitivity, as does the *mre11∆ exo1∆* double mutant (Figure 7B). Similar results have been reported for *rad50∆ exo1∆* (Tomita *et al.* 2003). The *exo1∆ rad8-LD* with a mutation in the E3 ligase domain was also more sensitive, whereas *exo1∆ rad8-HD* resembles *exo1∆* alone. Finally, the *exo1∆ rad8∆* and *exo1∆ rad8-LD* double mutants also showed increased sensitivity to hydroxyurea. These data suggest a role for Rad8 ubiquitin ligase in early stages of fork resection.

Long-range resection in budding yeast has also been linked to the ScSgs1 helicase and the Dna2 helicase/nuclease, in concert with RPA, and in opposition to fork regression (Cejka *et al.* 2010; H. Chen *et al.* 2013; Hu *et al.* 2012; Karanja *et al.* 2012; Niu *et al.* 2010). The corresponding pathway involved in fission yeast is reported to play only a minor role in resection (Langerak *et al.* 2011), but we examined interactions with mutations in *dna2* (Figure 7, C and D and Figure S4) and *rqh1* (Sc*SGS1*) (Figure 6F and Figure S4). The *dna2-K961T* mutant lacks helicase activity (Hu *et al.* 2012), and the single mutant is not sensitive to DNA-damaging drugs at the dosages used here. The *dna2-K961T rad8*Δ double mutant is sensitive to HU and even more so to MMS (Figure 7C). *dna2^ts^* has defective nuclease activity, and *dna2-K961T* complements its growth (Hu *et al.* 2012). The *dna2^ts^ rad8*Δ double mutant has slightly increased sensitivity to MMS (Figure 7D). In *S. pombe*, *rad11^+^* is the essential gene that encodes the largest subunit of the trimeric RPA (Parker *et al.* 1997). Even at permissive temperatures, *rad11A^ts^* is sensitive to all genotoxins tested. *rad8*Δ increased *rad11A^ts^* MMS and UV sensitivity, but partly rescued its HU sensitivity (Figure 7E ad Figure S4).

### Interactions with the fork protection complex

**Figure 8:**
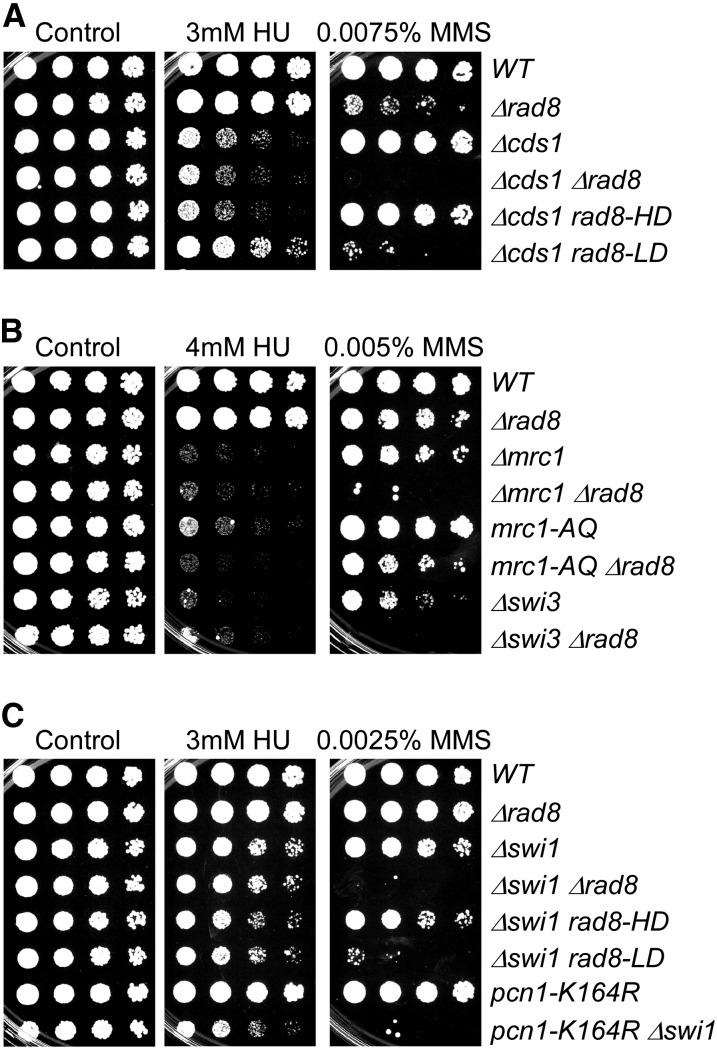
Rad8 works in parallel with replication checkpoint and fork protection complex to maintain fork stability. Strains were grown overnight at 32°, 1:5 serially diluted, and spotted to plain YES rich medium (Control) and YES with indicated drugs. Plates were incubated at 32° for 3 d.

Finally, we examined activities known to be involved with stabilization of the replication fork and resolution of stalled structures, which are required for MMS response (Figure 8 and Figure S4). The checkpoint kinase Cds1 physically interacts with Mus81 and regulates its function by phosphorylation-induced chromatin dissociation (Boddy *et al.* 2000; Kai *et al.* 2005). Both Mus81 and Cds1 contribute to slowing replication in the presence of MMS (Willis and Rhind 2009).

We observed that *cds1∆ rad8∆* mutants have increased sensitivity to MMS (Figure 8A). The *cds1∆ rad8∆* and *cds1∆ rad8-LD* showed a similar decline in viability, indicating that this sensitivity depends on the E3 ligase. Consistent with our other data, *cds1∆ rad8-HD* did not change the sensitivity of *cds1∆* on MMS. Curiously, *rad8-LD*, but not *rad8*∆ or *rad8-HD*, modestly rescued both *cds1*∆ CPT and HU sensitivity (Figure 8A and Figure S4).

Mrc1, Swi1, and Swi3 form the fork protection complex (FPC), which stabilizes the replication fork in the presence of stress and is required for MMS response (Noguchi *et al.* 2004; Sommariva *et al.* 2005). In addition to the function in fork protection, Mrc1 is also a replication checkpoint adaptor protein that facilitates Cds1 activation mediated by Rad3-Rad26 (Tanaka and Russell 2001; Zhao and Russell 2004; Zhao *et al.* 2003). The replication checkpoint activity of Mrc1 is abolished in *mrc1-AQ* mutant (Xu *et al.* 2006). Deletion of *rad8* increased the sensitivity of *swi1*∆, *swi3*∆, and *mrc1*∆ to MMS and UV (Figure 7, B and C and Figure S4). Loss of Rad8 ubiquitin ligase activity was the major contributor to these phenotypes. Consistent with this, there was a strong synthetic phenotype of *pcn1-K164R swi1∆*, consistent with the primary effect being through the ubiquitin ligase–mediated modification of PCNA.

## Discussion

Several lines of evidence implicate the HTLF/Rad5 family of proteins in helicase or translocase function. Human HLTF is capable of displacing RPA and PCNA from a modeled replication fork *in vitro* and promotes fork regression (Achar *et al.* 2011; Blastyak *et al.* 2010). Recent evidence suggests it can promote formation of a D-loop independent of Rad51 or Rad54 (Burkovics *et al.* 2014). Helicase activity associated with ScRad5 is thought to promote fork regression downstream of PCNA ubiquitylation in the error-free PRR pathway (Blastyak *et al.* 2007; Minca and Kowalski 2010). *Sc*Rad5 helicase is also required to restrain duplication-associated rearrangements (Putnam *et al.* 2010). Mutations that disrupt the ATP binding site in *ScRAD5* result in sensitivity to DNA damage (Chen *et al.* 2005; Minca and Kowalski 2010). However, in contrast to *S. cerevisiae rad5* (Kapitzky *et al.* 2010; Kats *et al.* 2009), *S. pombe rad8∆* mutants show no HU sensitivity (Figure 1A). This suggests that Rad8 is not required for response to, or recovery from, HU in otherwise wild-type cells; this may reflect the different requirements for HU response in these two fungi (Sabatinos *et al.* 2012).

We used a structure-function analysis to examine three conserved domains in *S. pombe rad8^+^*. Deletion of the HIRAN domain (found specifically in this family of proteins) disrupted nuclear localization and damage response *in vivo*. We restored localization by adding the nuclear localization sequence from SV40 large T antigen, but this did not restore the normal DNA damage response (Figure 2). The simplest conclusion is that nuclear localization is necessary but not sufficient for Rad8 function and the HIRAN domain makes a unique contribution to the damage response, although we cannot eliminate the possibility that our deletion has an indirect effect that disrupts protein structure.

We mutated the same residues as those shown in *ScRAD5* to disrupt the highly conserved ATP-binding site of the SNF2-related helicase domain and the E3 ubiquitin ligase domain. (Chen *et al.* 2005; Minca and Kowalski 2010; Ulrich 2003). The strongest effects observed were linked to the E3-ligase domain mutation (Figure 3). Although the null was worse in some conditions, the *rad8-HDLD* mutant generally resembled the *rad8-LD* mutant, suggesting that there may be a noncatalytic, structural role associated with the physical presence of the protein. We found that the mutation of the E3-ligase domain (*rad8-LD*) was responsible for all of the damage sensitivity observed in *rad8∆* single mutants. We found no evidence for the helicase domain functioning in otherwise wild-type cells.

We used the *rad8* point mutations as separation-of-function alleles to see which domains were required for the phenotypes. Epistasis with other components of the PRR pathway gave results as expected for Rad8 playing an essential role in PCNA ubiquitylation. The phenotype of *pcn1-K164R rad8∆* and *pcn1-K164R rad8-LD* on MMS were only slightly worse than the single mutant (Figure 4), suggesting that the predominant role of Rad8 ubiquitin ligase is via the Pcn1 K164 residue. Curiously, however, the *pcn1-K164R rad8-HD* double mutant was more sensitive to MMS than *pcn1-K164R rad8∆* or *pcn1-K164R* alone, and showed a slight sensitivity to HU. Thus, there is a more complex genetic interaction between *rad8* and *pcn1* mutants than would be suggested by a simple linear epistasis model, but deciphering this exceeds the generally qualitative nature of serial dilution assays.

We screened candidate helicases to assess any evidence for functional overlap with Rad8. A panel of strains from the Bioneer collection or our collection, which disrupted nonessential helicases, was “fingerprinted” for damage sensitivity (Figure 6, Figure 7, Figure S4, and Figure S5). Some candidates are missing from the version of the Bioneer collection we used and are not represented here. A substantial number of the mutants had no sensitivity to the agents we tested (HU, MMS, CPT, and UV), either alone or in combination with *rad8∆*, and were not investigated further. Of the four remaining phenotypic groups, the first group is defined by *rad8∆*, and is sensitive primarily to MMS. It contains one other helicase mutant, *rhp26∆*, required for transcription-coupled repair (Kanamitsu and Ikeda 2011). Not surprisingly, a double mutant *rad8∆ rhp26∆* showed dramatically increased sensitivity. The second group was only sensitive to CPT, an agent that causes S-phase–specific damage due to covalent coupling of topoisomerase to the ends of DNA (Wan *et al.* 1999). These mutants, which include several chromatin remodeling proteins, showed no synthetic interaction with *rad8∆*. Group 3 is represented by *srs2∆*, *snf22∆*, and a putative RNA helicase, and is only sensitive to CPT and HU, which cause defects specifically during S phase. We did not observe MMS sensitivity under these conditions with this allele of *snf22∆*, although there are other alleles that are reportedly MMS-sensitive (Dolan *et al.* 2010).

We observed a mixed interaction between *rad8∆* and *srs2∆* or *snf22∆*, in which MMS and CPT sensitivity were increased relative to the single mutants, but HU sensitivity was suppressed (Figure 6E and Figure S4), suggesting different roles for Rad8 in response to HU and MMS in these mutant backgrounds. The fourth group includes recombination-associated activities that are associated with replication fork restart. *chl1∆* has genetic interactions with an alternative RFC complex and with the fork protection complex (Ansbach *et al.* 2008); the double mutant increases MMS sensitivity but decreases CPT sensitivity (Figure S4). The other mutants define genes linked to HR and replication fork stability (Figure 6, B and F, Figure S4, and Figure S5): Fml1 promotes recombination at stalled forks while preventing crossovers (Nandi and Whitby 2012; Sun *et al.* 2008); Fbh1 directly antagonizes Rad51 (Lorenz *et al.* 2009) and Rqh1 limits Rad51-mediated fork restart (Lambert *et al.* 2010). In contrast to these activities limiting recombination, Rad54 promotes Rad51-mediated strand invasion (Muris *et al.* 1997; Heyer *et al.* 2010) and stimulates fork regression (Bugreev *et al.* 2011).

There is a striking growth defect in *rad51∆ rad8∆* strains that is not observed in double mutants with *rad54∆*, *rad55∆*, or *rad57∆* (Figure 6, A–D). The sensitivity in *rad51∆* can be attributed to the ubiquitin ligase domain, because *rad51∆ rad8-LD*, but not *rad51∆ rad8-HD*, showed increased sensitivity on HU. Because *rad51∆* mutants show evidence of genome instability and DNA damage in the absence of damaging agents (Nakamura *et al.* 2008; Sabatinos *et al.* 2012), these results suggest that Rad8 ubiquitin ligase activity is important to respond to the intrinsic stress in *rad51∆* mutants.

Double mutants between *rad8∆* and *rad54∆*, *rad55∆*, or *rad57∆* all had severe synthetic drug sensitivity to MMS treatment, and the same was observed for *rad8-LD* (Figure 6, A–D), which again was consistent with the central role of PCNA ubiquitylation in response to alkylation damage. However, the *rad8-HD* double mutants show modestly increased sensitivity relative to the HR single mutants, showing that there is a subtle contribution from the helicase domain when HR is affected.

Curiously, the results in HU were different for each of the separation of function alleles, suggesting that there are distinct differences between them. *rad54∆ rad8-LD* and *rad54∆ rad8-HD* had similar levels of sensitivity, which was almost as pronounced as *rad54∆ rad8∆* (Figure 6B), suggesting there may be a structural role or function for the HIRAN domain even in the absence of a catalytic activity. We conclude that both catalytic domains of Rad8 are important in the absence of Rad54 in HU, which could implicate Rad8 helicase as an alternative mechanism to Rad54 activity in HU. The *rad57∆* mutant was similar to *rad54∆*, although the degree of synthetic sensitivity was not as dramatic (Figure 6D). Surprisingly, because Rad55 and Rad57 work together (Liu *et al.* 2011; Sung 1997), *rad55∆ rad8-LD* and *rad55∆ rad8∆* were both HU-sensitive but *rad55∆ rad8-HD* was not (Figure 6C). Together, these data suggest a role for the Rad8 helicase domain in the absence of the typical Rad51 recombination pathway, which may be related to the capacity of the human HLTF protein to produce D-loops in a Rad51-independent and Rad54-independent pathway (Burkovics *et al.* 2014). They also suggest that the Rad55 and Rad57 proteins may not contribute equally to replication fork restart in HU.

Given these results in HU, we examined other mutations that disrupt replication fork restart. Srs2 contributes to fork reversal in repetitive sequences (Kerrest *et al.* 2009), and to fork restart and template switching at stalled forks (Lambert *et al.* 2010; Lorenz *et al.* 2009). On MMS, there was no synthetic interaction in *srs2∆ rad8∆* double mutants (Figure 6E). However, *rad8-HD* and *rad8∆* strongly rescued *srs2∆* when treated with HU. Because Srs2 antagonizes recombination, the opposite effects on HU sensitivity in the absence of *rad8* are consistent with Rad8 promoting HR, and opposing Srs2. This would be consistent with the modest *increase* in sensitivity in the *rad54∆ rad8* mutants. We propose that the helicase domain of Rad8 plays a minor role in Rad51-independent replication fork restart, particularly in HU.

The FANCM homolog Fml1 is already characterized as a translocase that regulates recombination at stalled forks (Nandi and Whitby 2012; Sun *et al.* 2008), and ICL repair by FANCM/ScMph1 depends on ScRad5 ubiquitin ligase activity (Daee *et al.* 2012). We observed synthetic phenotypes between *rad8∆* and *fml1∆* and showed these were linked to the E3-ligase domain (Figure S5). Similarly, double mutants between fork protection complex proteins and *rad8∆* showed strikingly increased MMS sensitivity that depended on the E3 ligase domain and the PCNA K164 residue that is ubiquitylated. Again, phenotypes on HU suggest that in this condition, both Rad8 domains make a modest contribution to survival.

The requirement for the ubiquitin ligase activity of Rad8 is consistent with evidence that PCNA is required at multiple points of repair (Frampton *et al.* 2006). The modification of PCNA in the PRR pathway in budding yeast is linked to completion of replication (Branzei *et al.* 2004). The PRR pathway is also required to suppress gross chromosome rearrangements and repeat associated expansions in budding yeast (Daee *et al.* 2007; Motegi *et al.* 2006a; Putnam *et al.* 2010). PCNA also limits D-loop extension during recombination (Sebesta *et al.* 2013). During resection, PCNA promotes processivity of Exo1 (X. Chen *et al.* 2013) and promotes end-joining (Chen *et al.* 2005).

The initial stages of resection work through an MRN-Ctp1 complex (Limbo *et al.* 2007). In turn, this initial event promotes Exo1-mediated bulk resection (Mimitou and Symington 2008). We see no obvious phenotype in *mre11∆ rad8∆* (Figure 7A and Figure S4), and little additional phenotype of a double mutant combining *rad8∆* with the other components of the MRN complex suggest that they function in a common epistasis group. However, a strong MMS-sensitive phenotype was observed when *rad8∆* or *rad8-LD* was combined with *exo1∆* (Figure 7B). This implicates PCNA ubiquitylation in resection pathways independent of Exo1, perhaps indicating a contribution to early stages of resection. Recent work shows that the single-stranded DNA binding protein RPA also plays a significant role in promoting resection. We observe a pronounced sensitivity to MMS in a *rad11^ts^ rad8∆* double mutant (Figure 7E).

This study suggests that while the general pathways and enzymes for genome stability are similar across eukaryotes, the activities of specific DNA regulators have diverged within those modules. Among the SNF2-specific helicases, for example, metazoans have significantly expanded the family (Hauk and Bowman 2011). Our data suggest that the helicase function of *S. pombe* Rad8 is potentially required under specialized circumstances, as a minor redundant pathway with the HR proteins most notably in the restart of replication forks in HU, or in the absence of ubiquitin-PCNA. The primary function of this enzyme in fission yeast in the maintenance of genome stability appears to be due to its E3 ubiquitin ligase activity and the HIRAN domain, indicating a fundamental role for PCNA modification in preserving genomic integrity.

## Supplementary Material

Supporting Information
