# Supplementary material for: Essential Domains of Schizosaccharomyces pombe Rad8 Required for DNA Damage Response
Source: G3 (Bethesda). 2014 May 28;4(8):1373–84. doi: 10.1534/g3.114.011346 (PMC4132169; doi:10.1534/g3.114.011346)
Supplement: Supporting Information [file supp_4_8_1373__index.html]

Essential Domains of Schizosaccharomyces pombe Rad8 Required for DNA Damage Response — Supporting Information 

# Essential Domains of *Schizosaccharomyces pombe* Rad8 Required for DNA Damage Response

## Supporting Information for Ding and Forsburg, 2014

**Files in this Data Supplement:**

- Supporting Information - Figures S1-S6 and Tables S1-S4 (PDF, 2 MB)
- Table S1 - Yeast strains used in this study. (PDF, 83 KB)
- Table S2 - Plasmids used in this study. (PDF, 73 KB)
- Table S3 - An analysis of the drug sensitivity of non-essential helicase mutants. (PDF, 84 KB)
- Table S4 - A survey of *rad8* genetic interaction with helicase mutants on different drugs. (PDF, 95 KB)
- Figure S1 - S18 is not required for Rad8 DNA damage response. (PDF, 211 KB)
- Figure S2 - Overproduction of *rad8* in *Δrad8* has minor defects. (PDF, 639 KB)
- Figure S3 - Rad8 functions in the PRR pathway. (PDF, 310 KB)
- Figure S4 - A damage fingerprint of helicase mutants. (PDF, 589 KB)
- Figure S5 - Fml1 and Rad8 ligase domain are functionally redundant. (PDF, 606 KB)
- Figure S6 - A comparison of the sensitivity of homologues recombination mutants to HU. (PDF, 104 KB)
