# Supplementary material for: Essential Domains of Schizosaccharomyces pombe Rad8 Required for DNA Damage Response
Source: G3 (Bethesda). 2014 May 28;4(8):1373–84. doi: 10.1534/g3.114.011346 (PMC4132169; doi:10.1534/g3.114.011346)
Supplement: Supporting Information [file supp_g3.114.011346_011346SI.pdf]

G3 · Genes | Genomes | Genetics

Essential domains of *S. pombe* Rad8 required for DNA damage response

Lin Ding* and Susan L Forsburg*

*Program in Molecular & Computational Biology, University of Southern California
Los Angeles, CA 90089-2910

Corresponding Author: Susan L Forsburg

Address: Program in Molecular & Computational Biology, University of Southern California, 1050 Childs Way, Los Angeles, CA 90089-2910
Phone 213-740-7342
Email: forsburg@usc.edu

DOI: 10.1534/g3.114.011346

Table S1 Yeast strains used in this study

Strain number	*Genotype*	Source
FY11	*h- ade6-M210*	Our stock
FY528	*h+ his3-D1 ura4-D18 leu1-32 ade6-M210*	Our stock
FY527	*h- his3-D1 ura4-D18 leu1-32 ade6-M216*	Our stock
FY5627	*h- ∆rad8::hphMX his3-D1 ura4-D18 leu1-32 ade6-M210*	This study
FY5698	*h- ∆rad8::hphMX his3-D1 ura4-D18 leu1-32 ade6-M210*	This study
FY5625	*h+ ∆rad8::hphMX his3-D1 ura4-D18 leu1-32 ade6-M216*	This study
FY5699	*h+ ∆rad8::hphMX his3-D1 ura4-D18 leu1-32 ade6-M210*	This study
FY444	*h+ rad8-190 ura4-D18 leu1-32 ade6-704*	Our stock
FY1884	*h- smt-0 ∆rad51::ura4+ ura4-D18 leu1-32 ade6-M210*	Our stock
FY6785	*h- smt-0 ∆rad51::ura4+ ∆rad8::hphMX ura4-D18 leu1-32 ade6-M210*	This study
FY1866	*h- smt-0 ∆rad54::ura4+ ura4-D18 leu1-32 ade6-M216*	Our stock
FY6868	*h- smt-0 ∆rad54::ura4+ ∆rad8::hphMX ura4-D18 leu1-32 ade6-M216*	This study
FY1389	*h- smt-0 ∆rad55::ura4+ ura4-D18*	Our stock
FY6809	*h- smt-0 ∆rad55::ura4+ ∆rad8::hphMX ura4-D18 ade6-M216*	This study
FY3770	*h- smt-0 ∆rad57::ura4+ his3-D1 ura4-D18 leu1-32 ade6-M210*	Our stock
FY6790	*h- smt-0 ∆rad57::ura4+ ∆rad8::hphMX his3-D1 ura4-D18 leu1-32 ade6-M210*	This study
FY6397	*h- loxP-dna2-K961T-loxM3 ura4-D18 leu1-32 ade6-704*	AW525 (Hu et al., 2012)
FY6428	*h- loxP-dna2-K961T-loxM3 ∆rad8::hphMX his3-D1 ura4-D18 leu1-32 ade6-M216*	This study
FY6452	*h- dna2ts:ura4 ura4-D18*	This study (Hu et al., 2012)
FY6505	*h- dna2ts:ura4 ∆rad8::hphMX his3-D1 ura4-D18 ade6-M216*	This study
FY254	*h- ura4-D18 leu1-32 ade6-M210 can1-1*	Our stock
FY6314	*h- loxP-rad8^{+}-loxM3 ura4-D18 leu1-32 ade6-M210 can1-1*	This study
FY6322	*h- loxP-∆rad8-loxM3 ura4-D18 leu1-32 ade6-M210 can1-1*	This study
FY6316	*h- loxP-rad8-∆HIRAN-loxM3 ura4-D18 leu1-32 ade6-M210 can1-1*	This study
FY6514	*h- loxP-rad8∆HIRAN::SV40NLS-loxM3 ura4-D18 leu1-32 ade6-M210 can1-1*	This study
FY6516	*h- loxP-rad8∆HIRAN::rad8NLS-loxM3 ura4-D18 leu1-32 ade6-M210 can1-1*	This study
FY6518	*h- loxP-rad8-∆NLS-loxM3 ura4-D18 leu1-32 ade6-M210 can1-1*	This study
FY6520	*h- loxP-rad8-HIRAN-loxM3 ura4-D18 leu1-32 ade6-M210 can1-1*	This study
FY6318	*h- loxP-rad8-K535AT536A-loxM3 leu1-32 ade6-M210 can1-1*	This study
FY6320	*h- loxP-rad8-I879A-loxM3 ura4-D18 leu1-32 ade6-M210 can1-1*	This study
FY6284	*h- loxP-rad8-K535AT536AI879A-loxM3 ura4-D18 leu1-32 ade6-M210 can1-1*	This study
FY7012	*h- loxP-rad8^{+}-5FLAG::KanMX6 ura4-D18 leu1-32 ade6-M210 can1-1*	This study
FY6905	*h- loxP-rad8-K535AT536A-5FLAG::KanMX6 ura4-D18 leu1-32 ade6-M210 can1-1*	This study

FY6959	h- loxP-rad8-I879A- 5FLAG::KanMX6 ura4-D18 leu1-32 ade6-M210 can1-1	This study
FY5904	h- rad8-5FLAG::kanMX6 his3-D1 ura4-D18 leu1-32 ade6-M216	This study
FY3123	h- Δrhp18::ura4+ ura4-D18 leu1-32 ade6-704	Our stock
FY6617	h- Δrhp18::ura4+ Δrad8::hphMX his3-D1 ura4-D18 leu1-32 ade6-M704	This study
FY6628	h- Δmms2::leu2 his3-D1 ura4-D18 leu1-32 ade6-M210	Our stock
FY6619	h- Δmms2::leu2 Δrad8::hphMX his3-D1 ura4-D18 leu1-32 ade6-M210	This study
FY6929	h- pcn1-K164R::ura4+ his3-D1 ura4-D18 leu1-32 ade6-M210	This study
FY6115	h- pcn1-K164R::ura4+ Δrad8::hphMX his3-D1 ura4-D18 leu1-32 ade6-M210	This study
FY6816	h- pcn1-K164R::ura4+ loxP-rad8-K535AT536A-loxM3 his3-D1 ura4-D18 leu1-32 ade6-M210	This study
FY6875	h- pcn1-K164R::ura4+ loxP-rad8-I879A-loxM3 his3-D1 ura4-D18 leu1-32 ade6-M210	This study
FY5128	h- Δsrs2::kan his3-D1 ura4-D18 leu1-32 ade6-M210	Our stock
FY5744	h- Δsrs2::kan Δrad8::hphMX his3-D1 ura4-D18 leu1-32 ade6-M210	This study
FY4841	h- eso1::kanMX6 kpa1::bleMX6 rev3::hphMX6 his3-D1 ura4-D18 leu1-32 ade6-M216	Our stock
FY6863	h- eso1::kanMX6 kpa1::bleMX6 rev3::hphMX6 Δrad8::hphMX his3-D1 ura4-D18 leu1-32 ade6-M216	This study
FY5555	h- Δfml1::natMX4 his3-D1 ura4-D18 leu1-32	This study From MCW2080 (Sun et al., 2008)
FY6436	h- Δfml1::natMX4 Δrad8::hphMX his3-D1 ura4-D18 leu1-32 ade6-M216	This study
FY5587	h- Δfml2:kanMX6-Bioneer his3-D1 ura4-D18 leu1-32 ade6-M216	This study
FY5726	h- Δfml2:kanMX6-Bioneer Δrad8::hphMX his3-D1 ura4-D18 leu1-32 ade6-M216	This study
FY6936	h- Δfml1::natMX4 Δfml2::KanMX6 his3-D1 ura4-D18 leu1-32	This study From MCW2082 (Sun et al., 2008)
FY5717	h- Δfml1::natMX4 Δfml2::KanMX6 Δrad8::hphMX his3-D1 ura4-D18 leu1-32	This study
FY6764	h- Δfml1::natMX4 Δfml2::KanMX6 loxP-rad8-K535AT536A-loxM3 ura4-D18 leu1-32	This study
FY6759	h- Δfml1::natMX4 Δfml2::KanMX6 loxP-rad8-K535AT536AI879A-loxM3 ura4-D18 leu1-32 ade6-M210	This study
FY6766	h- Δfml1::natMX4 Δfml2::KanMX6 loxP-rad8-I879A-loxM3 his3-D1 ura4-D18 leu1-32	This study
FY6825	h- pcn1-K164R::ura4+ Δfml1::natMX4 Δfml2::KanMX6 loxP-rad8-K535AT536A-loxM3 his3-D1 ura4-D18 leu1-32 ade6-M210	This study
FY6826	h- pcn1-K164R::ura4+ Δfml1::natMX4 Δfml2::KanMX6 Δrad8::hphMX his3-D1 ura4-D18 leu1-32 ade6-M210	This study
FY6257	h- Δfml1::natMX4 his3-D1 ura4-D18 leu1-32 ade6-M216	This study From MCW2080 (Sun et al., 2008)
FY6941	h- pcn1-K164R::ura4+ Δfml1::natMX his3-D1 ura4-D18 leu1-32 ade6-M210	This study
FY6948	h- Δfml2::KanMX6 ura4-D18 leu1-32 ade6-M210	This study From MCW2082

			(Sun et al., 2008)
FY6946	h-	*pcn1-K164R::ura4+ ∆fml2::KanMX6 his3-D1 ura4-D18 leu1-32 ade6-M210*	This study
FY6932	h-	*pcn1-K164R::ura4+ ∆fml1::natMX4 ∆fml2::KanMX6 his3-D1 ura4-D18 leu1-32 ade6-M210*	This study
FY2732	h-	*∆rad32::kanMX ura4-D18 leu1-32 ade6-M210*	Our stock
FY5892	h-	*∆rad32::kanMX ∆rad8::hphMX his3-D1 ura4-D18 leu1-32 ade6-M216*	This study
FY2733	h-	*∆rad50::kanMX ura4-D18 leu1-32 ade6-M210*	Our stock
FY5888	h-	*∆rad50::kanMX ∆rad8::hphMX ura4-D18 leu1-32 ade6-M216*	This study
FY2734	h-	*∆nbs1::kanMX ura4-D18 leu1-32*	Our stock
FY5895	h-	*∆nbs1::kanMX ∆rad8::hphMX his3-D1 ura4-D18 leu1-32 ade6-M216*	This study
FY5428	h-	*∆exo1::ura4+ ura4-D18 ade6-M210*	Our stock
FY6141	h-	*∆exo1::ura4+ ∆rad8::hphMX ura4-D18 ade6-M210*	This study
FY6820	h-	*∆exo1::ura4+ loxP-rad8-K535AT536A-loxM3 his3-D1 ura4-D18 leu1-32 ade6-M216*	This study
FY6879	h-	*∆exo1::ura4+ loxP-rad8-I879A-loxM3 his3-D1 ura4-D18 leu1-32 ade6-M216*	This study
FY790	h-	*rad11A-ts ura4-D18 leu1-32 ade6-M216*	Our stock
FY6797	h-	*rad11A-ts ∆rad8::hphMX his3-D1 ura4-D18 leu1-32 ade6-M210*	This study
FY3288	h-	*∆mus81::KanMX ura4-D18 ade6-M210*	Our stock
FY6118	h-	*∆mus81::KanMX ∆rad8::hphMX his3-D1 ura4-D18 ade6-M210*	This study
FY865	h-	*∆cds1::ura4 ura4-D18 leu1-32*	Our stock
FY5739	h-	*∆cds1::ura4+ ∆rad8::hphMX ura4-D18 leu1-32*	This study
FY6906	h-	*∆cds1::ura4 loxP-rad8-K535AT536A-loxM3 ura4-D18 leu1-32 ade6-M216*	This study
FY6897	h-	*∆cds1::ura4 loxP-rad8-I879A-loxM3 ura4-D18 leu1-32 ade6-M216*	This study
FY4685	h-	*∆mrc1::kanMX6-Bioneer his3-D1 ura4-D18 leu1-32 ade6-?*	Our stock
FY5742	h-	*∆mrc1::kanMX6-Bioneer ∆rad8::hphMX his3-D1 ura4-D18 leu1-32 ade6-M210*	This study
FY3529	h+	*∆mrc1::ura4+ leu1+::(mrc1(all S/TQ to AQ)-3HA) ura4-D18 ade6?*	Our stock
FY5885	h-	*∆mrc1::ura4+ leu1+::(mrc1(all S/TQ to AQ)-3HA) ∆rad8::hphMX his3-D1 ura4-D18 ade6-M210*	This study
FY3229	h-	*∆swi3::KanMX ura4-D18 leu1-32 ade6-M210*	Our stock
FY5784	h-	*∆swi3::KanMX ∆rad8::hphMX his3-D1 ura4-D18 leu1-32 ade6-M210*	This study
FY3226	h-	*∆swi1::kanMX his3-D1 ura4-D18 leu1-32 ade6-M210*	Our stock
FY5783	h-	*∆swi1::KanMX ∆rad8::hphMX his3-D1 ura4-D18 leu1-32 ade6-M210*	This study
FY6812	h-	*∆swi1::KanMX loxP-rad8-K535AT536A-loxM3 his3-D1 ura4-D18 leu1-32 ade6-M210*	This study
FY6871	h-	*∆swi1::KanMX loxP-rad8-I879A-loxM3 ura4-D18 leu1-32 ade6-M210*	This study
FY6821	h-	*∆swi1::kanMX pcn1-K164R::ura4 his3-D1 ura4-D18 leu1-32 ade6-M210*	This study
FY6400	h-	*∆rad8::hphMX his3-D1 ura4-D18 ade6-M210*	This study
FY6370	h-	*∆rad8::hphMX leu1-32::(nmt1-rad8-K535AT536A-GFP-leu1+) his3-D1 ura4-D18 ade6-M210*	This study
FY6372	h-	*∆rad8::hphMX leu1-32::(nmt1-rad8-I879A-GFP-leu1+) his3-D1 ura4-D18 ade6-M210*	This study
FY6374	h-	*∆rad8::hphMX leu1-32::(nmt1-rad8-K535AT536I879A-GFP-leu1+) his3-D1 ura4-D18 ade6-M210*	This study
FY6402	h-	*∆rad8::hphMX leu1-32::(nmt1-rad8-∆HIRAN-GFP-leu1+) his3-D1 ura4-D18 ade6-M210*	This study
FY6522	h-	*∆rad8::hphMX leu1-32::(nmt1-rad8∆HIRAN::SV40NLS-GFP-leu1+) his3-D1 ura4-D18 ade6-M210*	This study
FY6524	h-	*∆rad8::hphMX leu1-32::(nmt1-rad8∆HIRAN::rad8NLS-GFP-leu1+) his3-D1 ura4-D18 ade6-M210*	This study

FY6526	*h- Δrad8::hphMX leu1-32::(nmt1-rad8-no-NLS-GFP-leu1+) his3-D1 ura4-D18 ade6-M210*	This study
FY6528	*h- Δrad8::hphMX leu1-32::(nmt1-rad8-HIRAN-GFP-leu1+) his3-D1 ura4-D18 ade6-M210*	This study
FY3779	*h- Δfbh1::kanMX leu1-32 ura4-D18*	Our stock
FY5731	*h- Δfbh1::kanMX Δrad8::hphMX ura4-D18 leu1-32 ade6-M216*	This study
FY5745	*h- Δtlh2::kanMX6-Bioneer his3-D1 ura4-D18 leu1-32 ade6-M210*	This study
FY5746	*h+ Δtlh2::kanMX6-Bioneer Δrad8::hphMX leu1-32 ura4-D18 ade6-M216*	This study
FY5747	*h- Δswr1::kanMX6-Bioneer his3-D1 ura4-D18 leu1-32 ade6-M210*	This study
FY5748	*h- Δswr1::kanMX6-Bioneer Δrad8::hphMX his3-D1 ura4-D18 leu1-32 ade6-M210*	This study
FY5749	*h- ΔSPAC144.05::kanMX6-Bioneer leu1-32 ura4-D18 ade6-M210*	This study
FY5750	*h+ ΔSPAC144.05::kanMX6-Bioneer Δrad8::hphMX leu1-32 ura4-D18 ade6-M210*	This study
FY5751	*h+ Δrrp1::kanMX6-Bioneer leu1-32 ura4-D18 ade6-M210*	This study
FY5752	*h- Δrrp1::kanMX6-Bioneer Δrad8::hphMX his3-D1 ura4-D18 leu1-32 ade6-M210*	This study
FY5753	*h+ Δrdh54::kanMX6-Bioneer his3-D1 ura4-D18 leu1-32 ade6-M216*	This study
FY5754	*h- Δrdh54::kanMX6-Bioneer Δrad8::hphMX leu1-32 ura4-D18 ade6-M210*	This study
FY5755	*h- Δchl1::kanMX6-Bioneer leu1-32 ura4-D18 ade6-M210*	This study
FY5756	*h+ Δchl1::kanMX6-Bioneer Δrad8::hphMX leu1-32 ura4-D18 ade6-M210*	This study
FY5757	*h- ΔSPAC694.02::kanMX6-Bioneer leu1-32 ura4-D18 ade6-M210*	This study
FY5758	*h+ ΔSPAC694.02::kanMX6-Bioneer Δrad8::hphMX his3-D1 ura4-D18 leu1-32 ade6-M216*	This study
FY5759	*h- ΔSPBC15C4.05::kanMX6-Bioneer his3-D1 ura4-D18 leu1-32 ade6-M210*	This study
FY5760	*h+ ΔSPBC15C4.05::kanMX6-Bioneer Δrad8::hphMX his3-D1 ura4-D18 leu1-32 ade6-M216*	This study
FY5761	*h+ Δrrp2::kanMX6-Bioneer leu1-32 ura4-D18 ade6-M210*	This study
FY5762	*h- Δrrp2::kanMX6-Bioneer Δrad8::hphMX leu1-32 ura4-D18 ade6-M216 his3-D1*	This study
FY5763	*h+ Δhrp1::kanMX6-Bioneer leu1-32 ura4-D18 ade6-M216 his3-D1*	This study
FY5764	*h+ Δhrp1::kanMX6-Bioneer Δrad8::hphMX leu1-32 ura4-D18 ade6-M210*	This study
FY5765	*h- Δrhp26::kanMX6-Bioneer leu1-32 ura4-D18 ade6-M210*	This study
FY5766	*h+ Δrhp26::kanMX6-Bioneer Δrad8::hphMX leu1-32 ura4-D18 ade6-M216*	This study
FY5767	*h+ Δirc3::kanMX6-Bioneer leu1-32 ura4-D18 ade6-M210*	This study
FY5768	*h- Δirc3::kanMX6-Bioneer Δrad8::hphMX his3-D1 ura4-D18 leu1-32 ade6-M216*	This study
FY5769	*h- ΔSPBC582.10C::kanMX6-Bioneer his3-D1 ura4-D18 leu1-32 ade6-M210*	This study
FY5770	*h+ ΔSPBC582.10C::kanMX6-Bioneer Δrad8::hphMX ura4-D18 leu1-32 ade6-M210*	This study
FY5771	*h+ ΔSPCC737.07c::kanMX6-Bioneer his3-D1 ura4-D18 leu1-32 ade6-M216*	This study
FY5772	*h+ ΔSPCC737.07c::kanMX6-Bioneer Δrad8::hphMX ura4-D18 leu1-32 ade6-M210*	This study
FY5773	*h- Δhrp3::kanMX6-Bioneer his3-D1 leu1-32 ura4-D18 ade6-M216*	This study
FY5774	*h- Δhrp3::kanMX6-Bioneer Δrad8::hphMX ura4-D18 leu1-32 ade6-M210*	This study
FY4777	*h- Δsnf22::KanMX ura4-D18 leu1-32*	This study
FY5779	*h- Δsnf22::KanMX Δrad8::hphMX his3-D1 ura4-D18 leu1-32*	This study
FY4703	*h- pfh1-R20 leu1-32*	HT21 (Boule and Zakian, 2006)

FY6483	*h- pfh1-R20 Δrad8::hphMX ura4-D18 leu1-32 ade6-M216*	This study
FY6337	*h- Δrad8::hphMX ade6-M210*	This study
FY6434	*h- rad8-GFP::kanMX6 ade6-M210*	This study
FY2194	*h- Δrad3::ura4+ ura4-D18 ade6-M216*	Our stock
FY6315	*h- loxP-rad8^{+}-loxM3 leu1-32 ade6-M210 ura4-D18 can1-1*	This study
FY6317	*h- loxP-rad8-ΔHIRAN-loxM3 leu1-32 ade6-M210 ura4-D18 can1-1*	This study
FY6319	*h- loxP-rad8-K535AT536A-loxM3 leu1-32 ade6-M210 ura4-D18 can1-1*	This study
FY6321	*h- loxP-rad8-I879A-loxM3 leu1-32 ade6-M210 ura4-D18 can1-1*	This study
FY6284	*h- loxP-rad8-K535AT536AI879A-loxM3 leu1-32 ade6-M210 ura4-D18 can1-1*	This study
FY6323	*h- loxP-Δrad8-loxM3 leu1-32 ade6-M210 ura4-D18 can1-1*	This study
FY6394	*h- loxP-rad8-S18D-loxM3 leu1-32 ade6-M210 ura4-D18 can1-1*	This study
FY6399	*h- loxP-rad8-S18A-loxM3 leu1-32 ade6-M210 ura4-D18 can1-1*	This study

Table S2 Plasmids used in this study

Plasmid	Purpose	Source
pAW1	To construct *lox*-Cre base strain for *rad8*è FY5622	EUROSCARF: P30537
pAW8-XhoI	To make swap the *loxP loxM3* flanked region in the genome of FY5622	EUROSCARF: P30585
pLD45	To make swap the *loxP loxM3* flanked region in FY5622 with *rad8*$^+$	This study
pLD46	To make swap the *loxP loxM3* flanked region in FY5622 with *rad8-ΔHIRAN*	This study
pLD47	To make swap the *loxP loxM3* flanked region in FY5622 with *rad8-K535AT536A*	This study
pLD48	To make swap the *loxP loxM3* flanked region in FY5622 with *rad8-I879A*	This study
pLD49	To make swap the *loxP loxM3* flanked region in FY5622 with *rad8-K535AT536AI879A*	This study
pLD99	To make swap the *loxP loxM3* flanked region in FY5622 with *rad8-ΔHIRAN::SV40NLS*	This study
pLD100	To make swap the *loxP loxM3* flanked region in FY5622 with *rad8-ΔHIRAN::Rad8NLS*	This study
pLD101	To make swap the *loxP loxM3* flanked region in FY5622 with *rad8-ΔNLS*	This study
pLD102	To make swap the *loxP loxM3* flanked region in FY5622 with *rad8-HIRAN*	This study
pJK148	Integration at *leu1-32* locus	Our stock
pLD52	To integrate *rad8*$^+$*-GFP* into *leu1-32* locus	This study
pLD54	To integrate *rad8-K535AT536A-GFP* into *leu1-32* locus	This study
pLD55	To integrate *rad8-I879A-GFP* into *leu1-32* locus	This study
pLD56	To integrate *rad8- K535AT536AI879A-GFP* into *leu1-32* locus	This study
pLD53	To integrate *rad8-ΔHIRAN-GFP* into *leu1-32* locus	This study
pLD96	To integrate *rad8-ΔHIRAN::SV40NLS-GFP* into *leu1-32* locus	This study
pLD96	To integrate *rad8-ΔHIRAN::Rad8NLS-GFP* into *leu1-32* locus	This study
pLD97	To integrate *rad8-ΔNLS-GFP* into *leu1-32* locus	This study
pLD98	To integrate *rad8-HIRAN-GFP* into *leu1-32* locus	This study

Table S3 An analysis of the drug sensitivity of non-essential helicase mutants

Group	genotype	orthologs	growth	HU	MMS	UV	CPT
1: MMS specific	Δ*rhp26*	hXRCC3<br>ScRad26	-	-	↓	-	↓
	Δ*rad8*	HLTF, SHPRH<br>ScRad8	-	-	↓↓	↓	-
2: response to protein barriers	Δ*hrp1*	hCHD1, 2<br>ScChd1	-	-	-	-	↓↓
	Δ*swr1*	hEP400, hSRCAP<br>ScSwr1	-	-	-	-	↓↓
	Δ*SPBC15C4.05#*	hDHX29	-	-	-	-	↓↓↓
3: regulates HR	Δ*snf22*	hSMARCA4<br>ScSth1, ScSnf2	-	↓↓	-	-	↓
	Δ*srs2*	ScSrs2	-	↓	-	-	↓
	Δ*SPAC694.02**	hDDX60, hDDX60L	-	↓↓	-	-	↓↓↓
4: HR-associated	Δ*chl1*	hFANCJ<br>ScChl1	-	↓	↓	↓	↓
	Δ*fml1*	hFANCM<br>ScMph1	-	↓	↓↓	↓	↓
	Δ*fbh1*	hFBXO18	-	↓	↓↓	↓↓	↓
	Δ*rqh1*	hWRN, hBLM<br>ScSgs1	-	↓↓	↓↓	↓↓↓	↓↓↓
	Δ*rad54*	hRAD54L<br>ScRad54	↓	↓↓	↓↓↓	↓↓	↓↓↓
	Δ*rad57*	hXRCC3<br>ScRad57	-	↓	↓↓	↓	↓↓↓
5: no phenotype	Δ*hrp3*	hCHD1, hCHD2<br>ScChd1	-	-	-	-	-
	Δ*fml2*	hFANCM<br>ScMph1	-	-	-	-	-
	Δ*rrp1*	hTTF2<br>ScUls1	-	-	-	-	-
	Δ*rrp2*	hHLTF<br>ScUls1	-	-	-	-	-
	Δ*SPBC3B8.12**	ScIrc3	-	-	-	-	-
	Δ*SPBC582.10C*	ScRad16	-	-	-	-	-
	Δ*rdh54*	hRAD54B<br>ScRdh54	-	-	-	-	-
	Δ*SPCC737.07c*	hIGHMBP2<br>ScHcs1	-	-	-	-	-
	Δ*tlh2*	NA	-	-	-	-	-
	Δ*SPAC144.05*	hSHPRH<br>ScIRC20	-	-	-	-	-
	Δ*rad55*	hRAD51B<br>ScRad55	-	-	-	-	-

The level of sensitivity is scored by the fitness on the drug plates. No difference from wildtype is labeled as “-”. The level of sickness is scored by number of “↓”. NA = not available. h = Homo sapiens. Sc = *Saccharomyces cerevisiae.* HR = Homologous recombination. # RNA/DNA helicase. * RNA helicase. SPBC3B8.12 = SPBC11C11.11c.

Table S4 A survey of *rad8* genetic interaction with helicase mutants on different drugs

	genotype	growth	HU	MMS	UV	CPT
no synthetic defects	*Δhrp3 Δrad8*	-	-	-	-	-
	Δtlh2 Δrad8	-	-	-	-	-
	Δhrp1 Δrad8	-	-	-	-	-
	Δswr1 Δrad8	-	-	-	-	-
	Δrdh54 Δrad8	-	-	-	-	-
	Δrrp1 Δrad8	-	-	-	-	-
	Δrrp2 Δrad8	-	-	-	-	-
	ΔSPBC3B8.12 Δrad8	-	-	-	-	-
	ΔSPBC15C4.05 Δrad8	-	-	-	-	-
	ΔSPBC582.10C Δrad8	-	-	-	-	-
	ΔSPAC694.02 Δrad8	-	-	↑[$]	-	-
	Δ SPCC737.07c Δrad8	-	-	-	-	-
	ΔSPAC144.05 Δrad8	-	-	-	-	-
	cdc21-M68 Δrad8	-	-	-	-	-
	cdc21-C84 Δrad8	-	-	-	-	-
Increased drug sensitivity	*Δrhp26 Δrad8*	-	-	↓↓	-	-
	Δfbh1 Δrad8	-	-	↓	↓	-
	Δrqh1 Δrad8	-	↓	↓↓	↓	ND
	Δrad54 Δrad8	↓	↓↓↓	↓↓	↓	↓
	Δrad55 Δrad8	↓	↓	↓↓	↓↓	↓↓
	Δrad57 Δrad8	-	↓	↓↓	↓	↓
	pfh1-R20 Δrad8	-	↑	↓	-	-
	dna2-K961TΔrad8	-	↓	↓↓	↓	ND
	dna2^{ts} *Δrad8*	-	-	↓	↓	-
	Δfml1 Δrad8	-	↓↓	↓↓	↓↓	↓↓
	Δfml2 Δrad8	-	-	↓	-	-
mixed drug sensitivity	*Δfml1 Δfml2 Δrad8*	-<br>elongated	↓↓	↓↓↓	↓↓	↑
	Δchl1 Δrad8	-	-	↓↓	-	↑
	Δsrs2 Δrad8	-	↑	↓	-	↓↓
	Δsnf22 Δrad8	-	↑	-	-	↓

The level of sensitivity is scored by the fitness on the drug plates. No difference from the either of the single mutants is labeled as "-". The level of increased drug sensitivity is scored by number of "↓". The level of reduced drug sensitivity is scored by number of "↑". ND = not determined. [$] one RNA helicase partially decreased the MMS sensitivity of rad8. SPBC3B8.12 = SPBC11C11.11c.

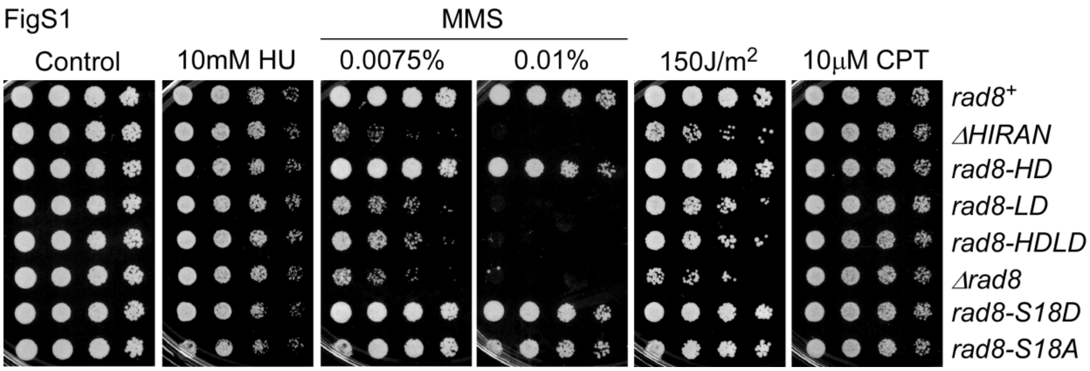

Figure S1 S18 is not required for Rad8 DNA damage response. Strains were grown overnight at 32ºC, 1:5 serially diluted and spotted to plain YES rich medium (Control) and YES with indicated drugs. Plates were incubated at 32ºC for 3 days.

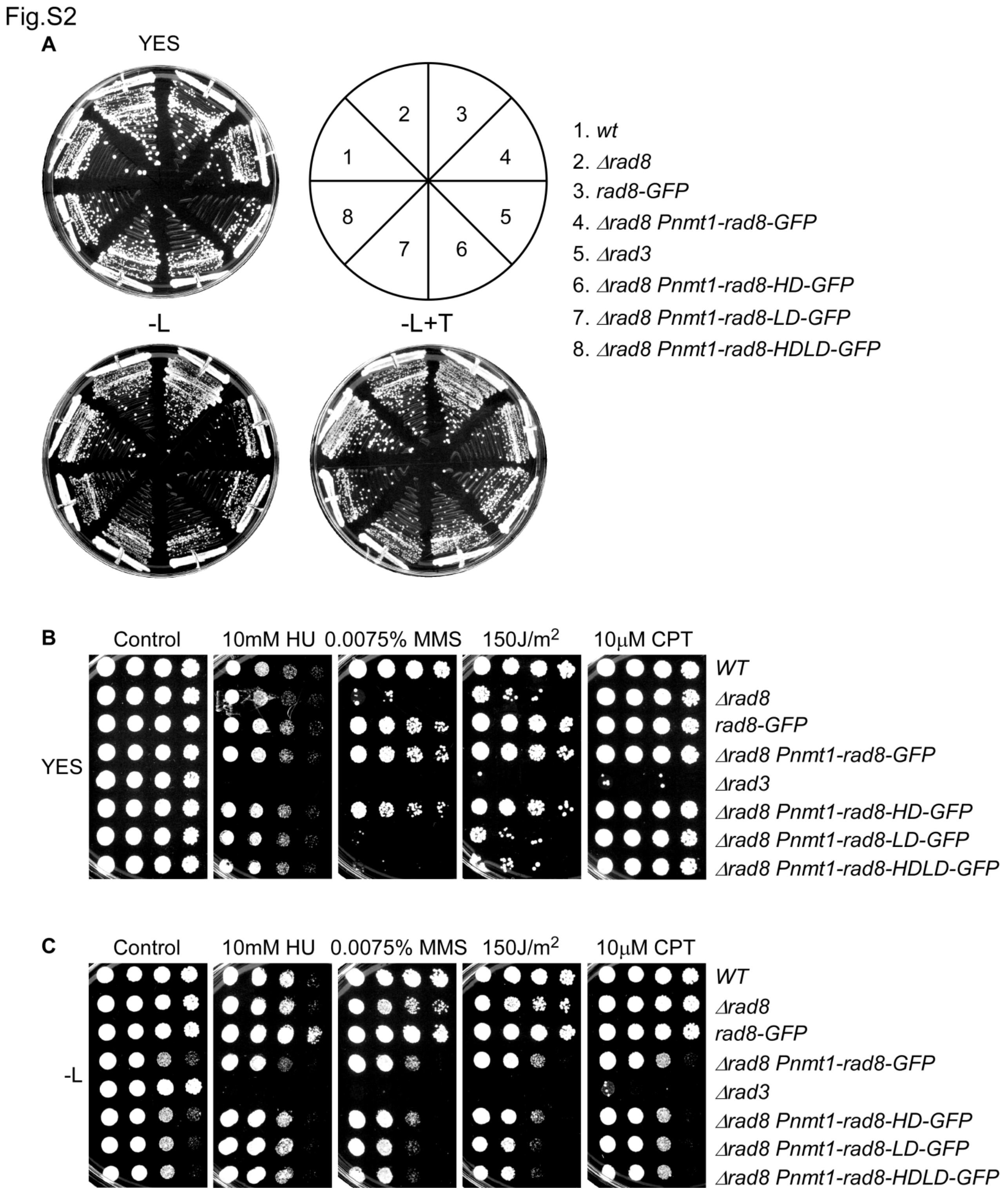

Figure S2 Overproduction of *rad8* in *Δrad8* has minor defects. (A) Overproduction (EMM-LEU plate) of *rad8-GFP* causes slightly growth defect. Strains were streaked out on different medium according to the schematics. (B) *rad8-GFP* fully complements Δ*rad8* and cells mount the same response to damaging drugs in the presence of thiamine (YES). Strains were grown overnight at 32ºC, 1:5 serially diluted and spotted to plain YES rich medium (Control) and YES with indicated drugs. (C) Overproduction of *rad8-GFP* and mutants is slightly toxic to cells. Strains were grown overnight at 32ºC, washed twice with EMM-LEU medium, 1:5 serially diluted in EMM-LEU and spotted to EMM-LEU medium (Control) and EMM-LEU with indicated drugs. Plates were incubated at 32ºC for 3 days if not indicated.

Fig.S3

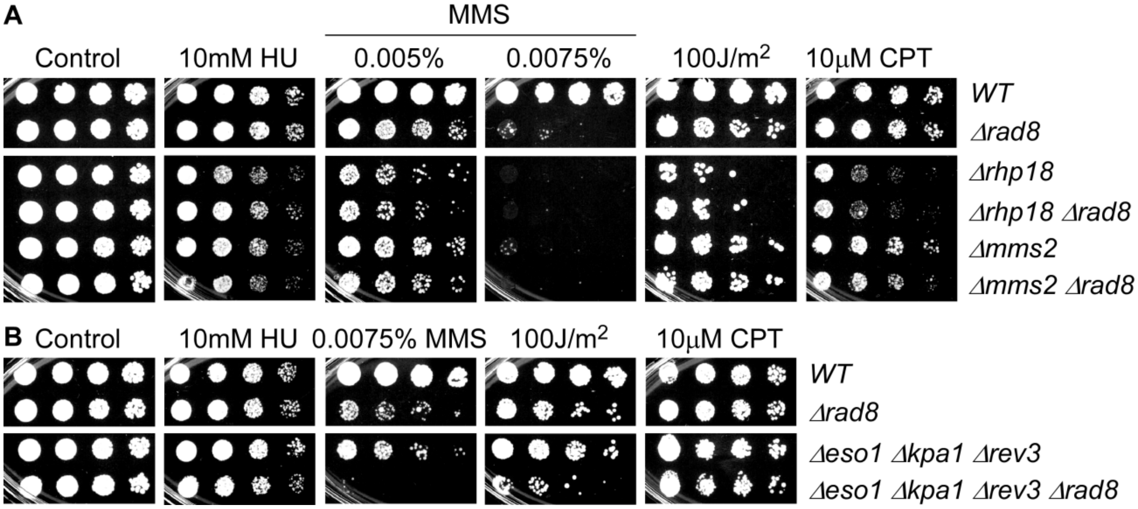

Figure S3 Rad8 functions in the PRR pathway. (A) *rad8*Δ is epistatic with *rhp18*Δ and *mms2*Δ. Strains were grown overnight at 32ºC, 1:5 serially diluted and spotted to plain YES rich medium (Control) and YES with indicated drugs. (B) *rad8*Δ is not epistatic with TLS mutants. Strains were grown overnight at 32ºC, 1:5 serially diluted and spotted to plain YES rich medium (Control) and YES with indicated drugs. Plates were incubated at 32ºC for 3 days.

Fig.S4

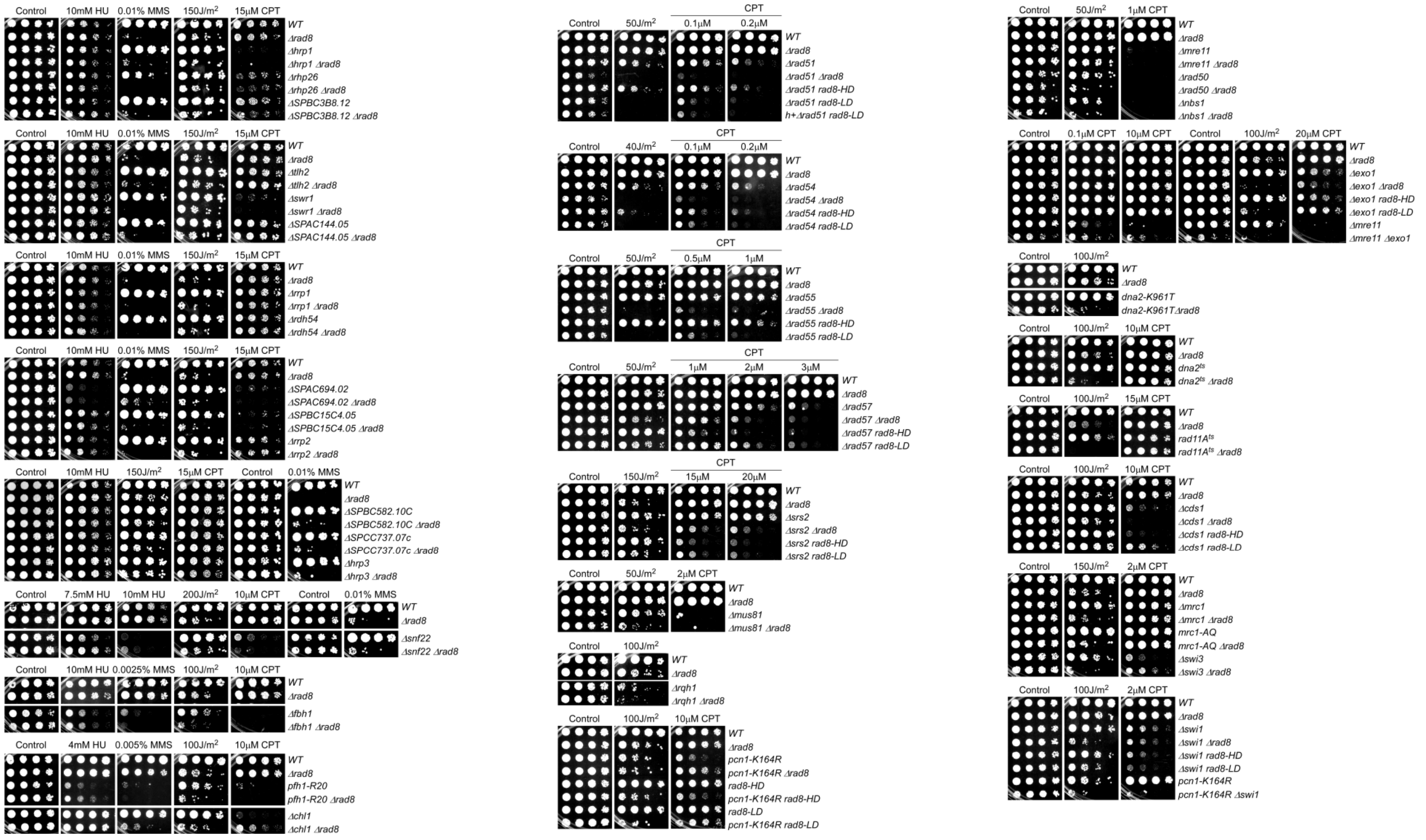

Figure S4 A damage fingerprint of helicase mutants Strains were grown overnight at 32ºC or 25ºC for ts strains, 1:5 serially diluted and spotted to plain YES rich medium (Control) and YES with indicated drugs. Plates were incubated at 32ºC for 3 days or 25ºC for 5 days.

Fig.S5

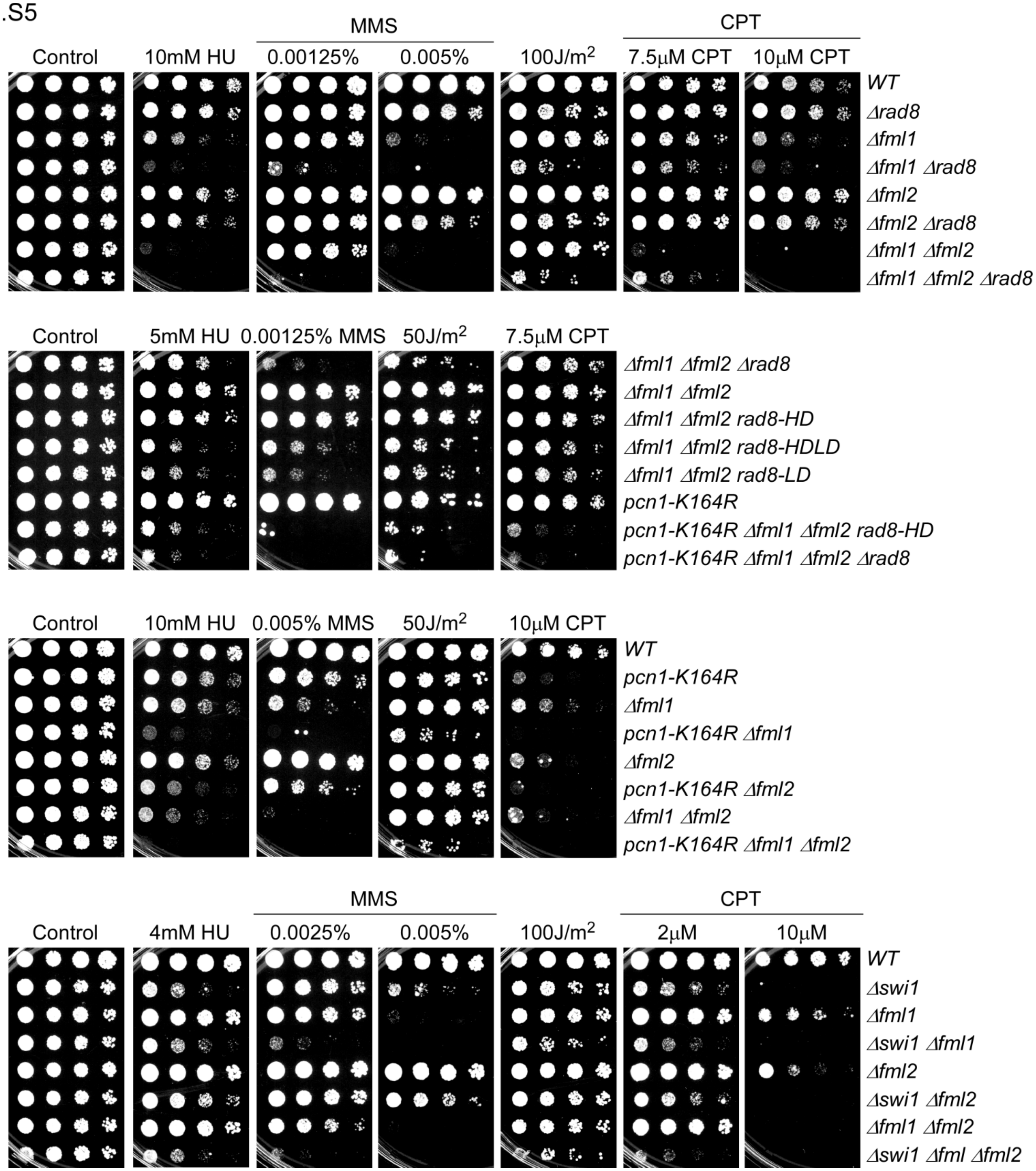

Figure S5 Fml1 and Rad8 ligase domain are functionally redundant. Strains were grown overnight at 32ºC, 1:5 serially diluted and spotted to plain YES rich medium (Control) and YES with indicated drugs. Plates were incubated at 32ºC for 3 days.

FigS6

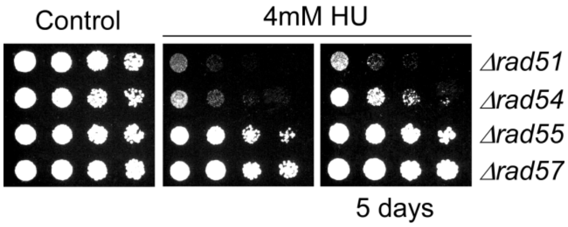

Figure S6 A comparison of the sensitivity of homologues recombination mutants to HU. Strains were grown overnight at 32ºC, 1:5 serially diluted and spotted to plain YES rich medium (Control) and YES with indicated drugs. Plates were incubated at 32ºC for 3 days unless otherwise indicated.
