# Supplementary material for: Essential Domains of Schizosaccharomyces pombe Rad8 Required for DNA Damage Response
Source: G3 (Bethesda). 2014 May 28;4(8):1373–84. doi: 10.1534/g3.114.011346 (PMC4132169; doi:10.1534/g3.114.011346)
Supplement: Supporting Information [file supp_g3.114.011346_TableS1.pdf]

Table S1 Yeast strains used in this study

Strain number	*Genotype*	Source
FY11	*h- ade6-M210*	Our stock
FY528	*h+ his3-D1 ura4-D18 leu1-32 ade6-M210*	Our stock
FY527	*h- his3-D1 ura4-D18 leu1-32 ade6-M216*	Our stock
FY5627	*h- ∆rad8::hphMX his3-D1 ura4-D18 leu1-32 ade6-M210*	This study
FY5698	*h- ∆rad8::hphMX his3-D1 ura4-D18 leu1-32 ade6-M210*	This study
FY5625	*h+ ∆rad8::hphMX his3-D1 ura4-D18 leu1-32 ade6-M216*	This study
FY5699	*h+ ∆rad8::hphMX his3-D1 ura4-D18 leu1-32 ade6-M210*	This study
FY444	*h+ rad8-190 ura4-D18 leu1-32 ade6-704*	Our stock
FY1884	*h- smt-0 ∆rad51::ura4+ ura4-D18 leu1-32 ade6-M210*	Our stock
FY6785	*h- smt-0 ∆rad51::ura4+ ∆rad8::hphMX ura4-D18 leu1-32 ade6-M210*	This study
FY1866	*h- smt-0 ∆rad54::ura4+ ura4-D18 leu1-32 ade6-M216*	Our stock
FY6868	*h- smt-0 ∆rad54::ura4+ ∆rad8::hphMX ura4-D18 leu1-32 ade6-M216*	This study
FY1389	*h- smt-0 ∆rad55::ura4+ ura4-D18*	Our stock
FY6809	*h- smt-0 ∆rad55::ura4+ ∆rad8::hphMX ura4-D18 ade6-M216*	This study
FY3770	*h- smt-0 ∆rad57::ura4+ his3-D1 ura4-D18 leu1-32 ade6-M210*	Our stock
FY6790	*h- smt-0 ∆rad57::ura4+ ∆rad8::hphMX his3-D1 ura4-D18 leu1-32 ade6-M210*	This study
FY6397	*h- loxP-dna2-K961T-loxM3 ura4-D18 leu1-32 ade6-704*	AW525 (Hu et al., 2012)
FY6428	*h- loxP-dna2-K961T-loxM3 ∆rad8::hphMX his3-D1 ura4-D18 leu1-32 ade6-M216*	This study
FY6452	*h- dna2ts:ura4 ura4-D18*	This study (Hu et al., 2012)
FY6505	*h- dna2ts:ura4 ∆rad8::hphMX his3-D1 ura4-D18 ade6-M216*	This study
FY254	*h- ura4-D18 leu1-32 ade6-M210 can1-1*	Our stock
FY6314	*h- loxP-rad8⁺-loxM3 ura4-D18 leu1-32 ade6-M210 can1-1*	This study
FY6322	*h- loxP-∆rad8-loxM3 ura4-D18 leu1-32 ade6-M210 can1-1*	This study
FY6316	*h- loxP-rad8-∆HIRAN-loxM3 ura4-D18 leu1-32 ade6-M210 can1-1*	This study
FY6514	*h- loxP-rad8∆HIRAN::SV40NLS-loxM3 ura4-D18 leu1-32 ade6-M210 can1-1*	This study
FY6516	*h- loxP-rad8∆HIRAN::rad8NLS-loxM3 ura4-D18 leu1-32 ade6-M210 can1-1*	This study
FY6518	*h- loxP-rad8-∆NLS-loxM3 ura4-D18 leu1-32 ade6-M210 can1-1*	This study
FY6520	*h- loxP-rad8-HIRAN-loxM3 ura4-D18 leu1-32 ade6-M210 can1-1*	This study
FY6318	*h- loxP-rad8-K535AT536A-loxM3 leu1-32 ade6-M210 can1-1*	This study
FY6320	*h- loxP-rad8-I879A-loxM3 ura4-D18 leu1-32 ade6-M210 can1-1*	This study
FY6284	*h- loxP-rad8-K535AT536AI879A-loxM3 ura4-D18 leu1-32 ade6-M210 can1-1*	This study
FY7012	*h- loxP-rad8⁺-5FLAG::KanMX6 ura4-D18 leu1-32 ade6-M210 can1-1*	This study
FY6905	*h- loxP-rad8-K535AT536A-5FLAG::KanMX6 ura4-D18 leu1-32 ade6-M210 can1-1*	This study

FY6959	h- loxP-rad8-I879A- 5FLAG::KanMX6 ura4-D18 leu1-32 ade6-M210 can1-1	This study
FY5904	h- rad8-5FLAG::kanMX6 his3-D1 ura4-D18 leu1-32 ade6-M216	This study
FY3123	h- ∆rhp18::ura4+ ura4-D18 leu1-32 ade6-704	Our stock
FY6617	h- ∆rhp18::ura4+ ∆rad8::hphMX his3-D1 ura4-D18 leu1-32 ade6-M704	This study
FY6628	h- ∆mms2::leu2 his3-D1 ura4-D18 leu1-32 ade6-M210	Our stock
FY6619	h- ∆mms2::leu2 ∆rad8::hphMX his3-D1 ura4-D18 leu1-32 ade6-M210	This study
FY6929	h- pcn1-K164R::ura4+ his3-D1 ura4-D18 leu1-32 ade6-M210	This study
FY6115	h- pcn1-K164R::ura4+ ∆rad8::hphMX his3-D1 ura4-D18 leu1-32 ade6-M210	This study
FY6816	h- pcn1-K164R::ura4+ loxP-rad8-K535AT536A-loxM3 his3-D1 ura4-D18 leu1-32 ade6-M210	This study
FY6875	h- pcn1-K164R::ura4+ loxP-rad8-I879A-loxM3 his3-D1 ura4-D18 leu1-32 ade6-M210	This study
FY5128	h- ∆srs2::kan his3-D1 ura4-D18 leu1-32 ade6-M210	Our stock
FY5744	h- ∆srs2::kan ∆rad8::hphMX his3-D1 ura4-D18 leu1-32 ade6-M210	This study
FY4841	h- eso1::kanMX6 kpa1::bleMX6 rev3::hphMX6 his3-D1 ura4-D18 leu1-32 ade6-M216	Our stock
FY6863	h- eso1::kanMX6 kpa1::bleMX6 rev3::hphMX6 ∆rad8::hphMX his3-D1 ura4-D18 leu1-32 ade6-M216	This study
		This study
FY5555	h- ∆fml1::natMX4 his3-D1 ura4-D18 leu1-32	From MCW2080 (Sun et al., 2008)
FY6436	h- ∆fml1::natMX4 ∆rad8::hphMX his3-D1 ura4-D18 leu1-32 ade6-M216	This study
FY5587	h- ∆fml2:kanMX6-Bioneer his3-D1 ura4-D18 leu1-32 ade6-M216	This study
FY5726	h- ∆fml2:kanMX6-Bioneer ∆rad8::hphMX his3-D1 ura4-D18 leu1-32 ade6-M216	This study
		This study
FY6936	h- ∆fml1::natMX4 ∆fml2::KanMX6 his3-D1 ura4-D18 leu1-32	From MCW2082 (Sun et al., 2008)
FY5717	h- ∆fml1::natMX4 ∆fml2::KanMX6 ∆rad8::hphMX his3-D1 ura4-D18 leu1-32	This study
FY6764	h- ∆fml1::natMX4 ∆fml2::KanMX6 loxP-rad8-K535AT536A-loxM3 ura4-D18 leu1-32	This study
FY6759	h- ∆fml1::natMX4 ∆fml2::KanMX6 loxP-rad8-K535AT536AI879A-loxM3 ura4-D18 leu1-32 ade6-M210	This study
FY6766	h- ∆fml1::natMX4 ∆fml2::KanMX6 loxP-rad8-I879A-loxM3 his3-D1 ura4-D18 leu1-32	This study
FY6825	h- pcn1-K164R::ura4+ ∆fml1::natMX4 ∆fml2::KanMX6 loxP-rad8-K535AT536A-loxM3 his3-D1 ura4-D18 leu1-32 ade6-M210	This study
FY6826	h- pcn1-K164R::ura4+ ∆fml1::natMX4 ∆fml2::KanMX6 ∆rad8::hphMX his3-D1 ura4-D18 leu1-32 ade6-M210	This study
		This study
FY6257	h- ∆fml1::natMX4 his3-D1 ura4-D18 leu1-32 ade6-M216	From MCW2080 (Sun et al., 2008)
FY6941	h- pcn1-K164R::ura4+ ∆fml1::natMX his3-D1 ura4-D18 leu1-32 ade6-M210	This study
		This study
FY6948	h- ∆fml2::KanMX6 ura4-D18 leu1-32 ade6-M210	From MCW2082

		(Sun et al., 2008)
FY6946	h- pcn1-K164R::ura4+ Δfml2::KanMX6 his3-D1 ura4-D18 leu1-32 ade6-M210	This study
FY6932	h- pcn1-K164R::ura4+ Δfml1::natMX4 Δfml2::KanMX6 his3-D1 ura4-D18 leu1-32 ade6-M210	This study
FY2732	h- Δrad32::kanMX ura4-D18 leu1-32 ade6-M210	Our stock
FY5892	h- Δrad32::kanMX Δrad8::hphMX his3-D1 ura4-D18 leu1-32 ade6-M216	This study
FY2733	h- Δrad50::kanMX ura4-D18 leu1-32 ade6-M210	Our stock
FY5888	h- Δrad50::kanMX Δrad8::hphMX ura4-D18 leu1-32 ade6-M216	This study
FY2734	h- Δnbs1::kanMX ura4-D18 leu1-32	Our stock
FY5895	h- Δnbs1::kanMX Δrad8::hphMX his3-D1 ura4-D18 leu1-32 ade6-M216	This study
FY5428	h- Δexo1::ura4+ ura4-D18 ade6-M210	Our stock
FY6141	h- Δexo1::ura4+ Δrad8::hphMX ura4-D18 ade6-M210	This study
FY6820	h- Δexo1::ura4+ loxP-rad8-K535AT536A-loxM3 his3-D1 ura4-D18 leu1-32 ade6-M216	This study
FY6879	h- Δexo1::ura4+ loxP-rad8-I879A-loxM3 his3-D1 ura4-D18 leu1-32 ade6-M216	This study
FY790	h- rad11A-ts ura4-D18 leu1-32 ade6-M216	Our stock
FY6797	h- rad11A-ts Δrad8::hphMX his3-D1 ura4-D18 leu1-32 ade6-M210	This study
FY3288	h- Δmus81::KanMX ura4-D18 ade6-M210	Our stock
FY6118	h- Δmus81::KanMX Δrad8::hphMX his3-D1 ura4-D18 ade6-M210	This study
FY865	h- Δcds1::ura4 ura4-D18 leu1-32	Our stock
FY5739	h- Δcds1::ura4+ Δrad8::hphMX ura4-D18 leu1-32	This study
FY6906	h- Δcds1::ura4 loxP-rad8-K535AT536A-loxM3 ura4-D18 leu1-32 ade6-M216	This study
FY6897	h- Δcds1::ura4 loxP-rad8-I879A-loxM3 ura4-D18 leu1-32 ade6-M216	This study
FY4685	h- Δmrc1::kanMX6-Bioneer his3-D1 ura4-D18 leu1-32 ade6-?	Our stock
FY5742	h- Δmrc1::kanMX6-Bioneer Δrad8::hphMX his3-D1 ura4-D18 leu1-32 ade6-M210	This study
FY3529	h+ Δmrc1::ura4+ leu1+::(mrc1(all S/TQ to AQ)-3HA) ura4-D18 ade6?	Our stock
FY5885	h- Δmrc1::ura4+ leu1+::(mrc1(all S/TQ to AQ)-3HA) Δrad8::hphMX his3-D1 ura4-D18 ade6-M210	This study
FY3229	h- Δswi3::KanMX ura4-D18 leu1-32 ade6-M210	Our stock
FY5784	h- Δswi3::KanMX Δrad8::hphMX his3-D1 ura4-D18 leu1-32 ade6-M210	This study
FY3226	h- Δswi1::kanMX his3-D1 ura4-D18 leu1-32 ade6-M210	Our stock
FY5783	h- Δswi1::KanMX Δrad8::hphMX his3-D1 ura4-D18 leu1-32 ade6-M210	This study
FY6812	h- Δswi1::KanMX loxP-rad8-K535AT536A-loxM3 his3-D1 ura4-D18 leu1-32 ade6-M210	This study
FY6871	h- Δswi1::KanMX loxP-rad8-I879A-loxM3 ura4-D18 leu1-32 ade6-M210	This study
FY6821	h- Δswi1::kanMX pcn1-K164R::ura4 his3-D1 ura4-D18 leu1-32 ade6-M210	This study
FY6400	h- Δrad8::hphMX his3-D1 ura4-D18 ade6-M210	This study
FY6370	h- Δrad8::hphMX leu1-32::(nmt1-rad8-K535AT536A-GFP-leu1+) his3-D1 ura4-D18 ade6-M210	This study
FY6372	h- Δrad8::hphMX leu1-32::(nmt1-rad8-I879A-GFP-leu1+) his3-D1 ura4-D18 ade6-M210	This study
FY6374	h- Δrad8::hphMX leu1-32::(nmt1-rad8-K535AT536I879A-GFP-leu1+) his3-D1 ura4-D18 ade6-M210	This study
FY6402	h- Δrad8::hphMX leu1-32::(nmt1-rad8-ΔHIRAN-GFP-leu1+) his3-D1 ura4-D18 ade6-M210	This study
FY6522	h- Δrad8::hphMX leu1-32::(nmt1-rad8ΔHIRAN::SV40NLS-GFP-leu1+) his3-D1 ura4-D18 ade6-M210	This study
FY6524	h- Δrad8::hphMX leu1-32::(nmt1-rad8ΔHIRAN::rad8NLS-GFP-leu1+) his3-D1 ura4-D18 ade6-M210	This study

FY6526	*h- Δrad8::hphMX leu1-32::(nmt1-rad8-no-NLS-GFP-leu1+) his3-D1 ura4-D18 ade6-M210*	This study
FY6528	*h- Δrad8::hphMX leu1-32::(nmt1-rad8-HIRAN-GFP-leu1+) his3-D1 ura4-D18 ade6-M210*	This study
FY3779	*h- Δfbh1::kanMX leu1-32 ura4-D18*	Our stock
FY5731	*h- Δfbh1::kanMX Δrad8::hphMX ura4-D18 leu1-32 ade6-M216*	This study
FY5745	*h- Δtlh2::kanMX6-Bioneer his3-D1 ura4-D18 leu1-32 ade6-M210*	This study
FY5746	*h+ Δtlh2::kanMX6-Bioneer Δrad8::hphMX leu1-32 ura4-D18 ade6-M216*	This study
FY5747	*h- Δswr1::kanMX6-Bioneer his3-D1 ura4-D18 leu1-32 ade6-M210*	This study
FY5748	*h- Δswr1::kanMX6-Bioneer Δrad8::hphMX his3-D1 ura4-D18 leu1-32 ade6-M210*	This study
FY5749	*h- ΔSPAC144.05::kanMX6-Bioneer leu1-32 ura4-D18 ade6-M210*	This study
FY5750	*h+ ΔSPAC144.05::kanMX6-Bioneer Δrad8::hphMX leu1-32 ura4-D18 ade6-M210*	This study
FY5751	*h+ Δrrp1::kanMX6-Bioneer leu1-32 ura4-D18 ade6-M210*	This study
FY5752	*h- Δrrp1::kanMX6-Bioneer Δrad8::hphMX his3-D1 ura4-D18 leu1-32 ade6-M210*	This study
FY5753	*h+ Δrdh54::kanMX6-Bioneer his3-D1 ura4-D18 leu1-32 ade6-M216*	This study
FY5754	*h- Δrdh54::kanMX6-Bioneer Δrad8::hphMX leu1-32 ura4-D18 ade6-M210*	This study
FY5755	*h- Δchl1::kanMX6-Bioneer leu1-32 ura4-D18 ade6-M210*	This study
FY5756	*h+ Δchl1::kanMX6-Bioneer Δrad8::hphMX leu1-32 ura4-D18 ade6-M210*	This study
FY5757	*h- ΔSPAC694.02::kanMX6-Bioneer leu1-32 ura4-D18 ade6-M210*	This study
FY5758	*h+ ΔSPAC694.02::kanMX6-Bioneer Δrad8::hphMX his3-D1 ura4-D18 leu1-32 ade6-M216*	This study
FY5759	*h- ΔSPBC15C4.05::kanMX6-Bioneer his3-D1 ura4-D18 leu1-32 ade6-M210*	This study
FY5760	*h+ ΔSPBC15C4.05::kanMX6-Bioneer Δrad8::hphMX his3-D1 ura4-D18 leu1-32 ade6-M216*	This study
FY5761	*h+ Δrrp2::kanMX6-Bioneer leu1-32 ura4-D18 ade6-M210*	This study
FY5762	*h- Δrrp2::kanMX6-Bioneer Δrad8::hphMX leu1-32 ura4-D18 ade6-M216 his3-D1*	This study
FY5763	*h+ Δhrp1::kanMX6-Bioneer leu1-32 ura4-D18 ade6-M216 his3-D1*	This study
FY5764	*h+ Δhrp1::kanMX6-Bioneer Δrad8::hphMX leu1-32 ura4-D18 ade6-M210*	This study
FY5765	*h- Δrhp26::kanMX6-Bioneer leu1-32 ura4-D18 ade6-M210*	This study
FY5766	*h+ Δrhp26::kanMX6-Bioneer Δrad8::hphMX leu1-32 ura4-D18 ade6-M216*	This study
FY5767	*h+ Δirc3::kanMX6-Bioneer leu1-32 ura4-D18 ade6-M210*	This study
FY5768	*h- Δirc3::kanMX6-Bioneer Δrad8::hphMX his3-D1 ura4-D18 leu1-32 ade6-M216*	This study
FY5769	*h- ΔSPBC582.10C::kanMX6-Bioneer his3-D1 ura4-D18 leu1-32 ade6-M210*	This study
FY5770	*h+ ΔSPBC582.10C::kanMX6-Bioneer Δrad8::hphMX ura4-D18 leu1-32 ade6-M210*	This study
FY5771	*h+ ΔSPCC737.07c::kanMX6-Bioneer his3-D1 ura4-D18 leu1-32 ade6-M216*	This study
FY5772	*h+ ΔSPCC737.07c::kanMX6-Bioneer Δrad8::hphMX ura4-D18 leu1-32 ade6-M210*	This study
FY5773	*h- Δhrp3::kanMX6-Bioneer his3-D1 leu1-32 ura4-D18 ade6-M216*	This study
FY5774	*h- Δhrp3::kanMX6-Bioneer Δrad8::hphMX ura4-D18 leu1-32 ade6-M210*	This study
FY4777	*h- Δsnf22::KanMX ura4-D18 leu1-32*	This study
FY5779	*h- Δsnf22::KanMX Δrad8::hphMX his3-D1 ura4-D18 leu1-32*	This study
FY4703	*h- pfh1-R20 leu1-32*	HT21 (Boule and Zakian, 2006)

FY6483	*h- pfh1-R20 Δrad8::hphMX ura4-D18 leu1-32 ade6-M216*	This study
FY6337	*h- Δrad8::hphMX ade6-M210*	This study
FY6434	*h- rad8-GFP::kanMX6 ade6-M210*	This study
FY2194	*h- Δrad3::ura4+ ura4-D18 ade6-M216*	Our stock
FY6315	*h- loxP-rad8⁺-loxM3 leu1-32 ade6-M210 ura4-D18 can1-1*	This study
FY6317	*h- loxP-rad8-ΔHIRAN-loxM3 leu1-32 ade6-M210 ura4-D18 can1-1*	This study
FY6319	*h- loxP-rad8-K535AT536A-loxM3 leu1-32 ade6-M210 ura4-D18 can1-1*	This study
FY6321	*h- loxP-rad8-I879A-loxM3 leu1-32 ade6-M210 ura4-D18 can1-1*	This study
FY6284	*h- loxP-rad8-K535AT536AI879A-loxM3 leu1-32 ade6-M210 ura4-D18 can1-1*	This study
FY6323	*h- loxP-Δrad8-loxM3 leu1-32 ade6-M210 ura4-D18 can1-1*	This study
FY6394	*h- loxP-rad8-S18D-loxM3 leu1-32 ade6-M210 ura4-D18 can1-1*	This study
FY6399	*h- loxP-rad8-S18A-loxM3 leu1-32 ade6-M210 ura4-D18 can1-1*	This study
