# Supplementary material for: Essential Domains of Schizosaccharomyces pombe Rad8 Required for DNA Damage Response
Source: G3 (Bethesda). 2014 May 28;4(8):1373–84. doi: 10.1534/g3.114.011346 (PMC4132169; doi:10.1534/g3.114.011346)
Supplement: Supporting Information [file supp_g3.114.011346_TableS2.pdf]

Table S2 Plasmids used in this study

Plasmid	Purpose	Source
pAW1	To construct *lox*-Cre base strain for *rad8*è FY5622	EUROSCARF: P30537
pAW8-XhoI	To make swap the *loxP loxM3* flanked region in the genome of FY5622	EUROSCARF: P30585
pLD45	To make swap the *loxP loxM3* flanked region in FY5622 with *rad8*$^+$	This study
pLD46	To make swap the *loxP loxM3* flanked region in FY5622 with *rad8-∆HIRAN*	This study
pLD47	To make swap the *loxP loxM3* flanked region in FY5622 with *rad8-K535AT536A*	This study
pLD48	To make swap the *loxP loxM3* flanked region in FY5622 with *rad8-I879A*	This study
pLD49	To make swap the *loxP loxM3* flanked region in FY5622 with *rad8-K535AT536AI879A*	This study
pLD99	To make swap the *loxP loxM3* flanked region in FY5622 with *rad8-∆HIRAN::SV40NLS*	This study
pLD100	To make swap the *loxP loxM3* flanked region in FY5622 with *rad8-∆HIRAN::Rad8NLS*	This study
pLD101	To make swap the *loxP loxM3* flanked region in FY5622 with *rad8-∆NLS*	This study
pLD102	To make swap the *loxP loxM3* flanked region in FY5622 with *rad8-HIRAN*	This study
pJK148	Integration at *leu1-32* locus	Our stock
pLD52	To integrate *rad8*$^+$*-GFP* into *leu1-32* locus	This study
pLD54	To integrate *rad8-K535AT536A-GFP* into *leu1-32* locus	This study
pLD55	To integrate *rad8-I879A-GFP* into *leu1-32* locus	This study
pLD56	To integrate *rad8- K535AT536AI879A-GFP* into *leu1-32* locus	This study
pLD53	To integrate *rad8-∆HIRAN-GFP* into *leu1-32* locus	This study
pLD96	To integrate *rad8-∆HIRAN::SV40NLS-GFP* into *leu1-32* locus	This study
pLD96	To integrate *rad8-∆HIRAN::Rad8NLS-GFP* into *leu1-32* locus	This study
pLD97	To integrate *rad8-∆NLS-GFP* into *leu1-32* locus	This study
pLD98	To integrate *rad8-HIRAN-GFP* into *leu1-32* locus	This study
