# Supplementary material for: Essential Domains of Schizosaccharomyces pombe Rad8 Required for DNA Damage Response
Source: G3 (Bethesda). 2014 May 28;4(8):1373–84. doi: 10.1534/g3.114.011346 (PMC4132169; doi:10.1534/g3.114.011346)
Supplement: Supporting Information [file supp_g3.114.011346_TableS3.pdf]

Table S3 An analysis of the drug sensitivity of non-essential helicase mutants

Group	genotype	orthologs	growth	HU	MMS	UV	CPT
1: MMS specific	Δ*rhp26*	hXRCC3<br>ScRad26	-	-	↓	-	↓
	Δ*rad8*	HLTF, SHPRH<br>ScRad8	-	-	↓↓	↓	-
2: response to protein barriers	Δ*hrp1*	hCHD1, 2<br>ScChd1	-	-	-	-	↓↓
	Δ*swr1*	hEP400, hSRCAP<br>ScSwr1	-	-	-	-	↓↓
	Δ*SPBC15C4.05#*	hDHX29	-	-	-	-	↓↓↓
3: regulates HR	Δ*snf22*	hSMARCA4<br>ScSth1, ScSnf2	-	↓↓	-	-	↓
	Δ*srs2*	ScSrs2	-	↓	-	-	↓
	Δ*SPAC694.02**	hDDX60, hDDX60L	-	↓↓	-	-	↓↓↓
4: HR-associated	Δ*chl1*	hFANCJ<br>ScChl1	-	↓	↓	↓	↓
	Δ*fml1*	hFANCM<br>ScMph1	-	↓	↓↓	↓	↓
	Δ*fbh1*	hFBXO18	-	↓	↓↓	↓↓	↓
	Δ*rqh1*	hWRN, hBLM<br>ScSgs1	-	↓↓	↓↓	↓↓↓	↓↓↓
	Δ*rad54*	hRAD54L<br>ScRad54	↓	↓↓	↓↓↓	↓↓	↓↓↓
	Δ*rad57*	hXRCC3<br>ScRad57	-	↓	↓↓	↓	↓↓↓
5: no phenotype	Δ*hrp3*	hCHD1, hCHD2<br>ScChd1	-	-	-	-	-
	Δ*fml2*	hFANCM<br>ScMph1	-	-	-	-	-
	Δ*rrp1*	hTTF2<br>ScUls1	-	-	-	-	-
	Δ*rrp2*	hHLTF<br>ScUls1	-	-	-	-	-
	Δ*SPBC3B8.12**	ScIrc3	-	-	-	-	-
	Δ*SPBC582.10C*	ScRad16	-	-	-	-	-
	Δ*rdh54*	hRAD54B<br>ScRdh54	-	-	-	-	-
	Δ*SPCC737.07c*	hIGHMBP2<br>ScHcs1	-	-	-	-	-
	Δ*tlh2*	NA	-	-	-	-	-
	Δ*SPAC144.05*	hSHPRH<br>ScIRC20	-	-	-	-	-
	Δ*rad55*	hRAD51B<br>ScRad55	-	-	-	-	-

The level of sensitivity is scored by the fitness on the drug plates. No difference from wildtype is labeled as "-". The level of sickness is scored by number of "↓". NA = not available. h = Homo sapiens. Sc = *Saccharomyces cerevisiae.* HR = Homologous recombination. # RNA/DNA helicase. * RNA helicase. SPBC3B8.12 = SPBC11C11.11c.
