# Supplementary material for: Essential Domains of Schizosaccharomyces pombe Rad8 Required for DNA Damage Response
Source: G3 (Bethesda). 2014 May 28;4(8):1373–84. doi: 10.1534/g3.114.011346 (PMC4132169; doi:10.1534/g3.114.011346)
Supplement: Supporting Information [file supp_g3.114.011346_TableS4.pdf]

Table S4 A survey of *rad8* genetic interaction with helicase mutants on different drugs

	genotype	growth	HU	MMS	UV	CPT
no synthetic defects	*Δhrp3 Δrad8*	-	-	-	-	-
	Δtlh2 Δrad8	-	-	-	-	-
	Δhrp1 Δrad8	-	-	-	-	-
	Δswr1 Δrad8	-	-	-	-	-
	Δrdh54 Δrad8	-	-	-	-	-
	Δrrp1 Δrad8	-	-	-	-	-
	Δrrp2 Δrad8	-	-	-	-	-
	ΔSPBC3B8.12 Δrad8	-	-	-	-	-
	ΔSPBC15C4.05 Δrad8	-	-	-	-	-
	ΔSPBC582.10C Δrad8	-	-	-	-	-
	ΔSPAC694.02 Δrad8	-	-	↑$^{\$}$	-	-
	Δ SPCC737.07c Δrad8	-	-	-	-	-
	ΔSPAC144.05 Δrad8	-	-	-	-	-
	cdc21-M68 Δrad8	-	-	-	-	-
	cdc21-C84 Δrad8	-	-	-	-	-
Increased drug sensitivity	*Δrhp26 Δrad8*	-	-	↓↓	-	-
	Δfbh1 Δrad8	-	-	↓	↓	-
	Δrqh1 Δrad8	-	↓	↓↓	↓	ND
	Δrad54 Δrad8	↓	↓↓↓	↓↓	↓	↓
	Δrad55 Δrad8	↓	↓	↓↓	↓↓	↓↓
	Δrad57 Δrad8	-	↓	↓↓	↓	↓
	pfh1-R20 Δrad8	-	↑	↓	-	-
	dna2-K961TΔrad8	-	↓	↓↓	↓	ND
	dna2^{ts} Δrad8	-	-	↓	↓	-
	Δfml1 Δrad8	-	↓↓	↓↓	↓↓	↓↓
	Δfml2 Δrad8	-	-	↓	-	-
mixed drug sensitivity	*Δfml1 Δfml2 Δrad8*	-<br>elongated	↓↓	↓↓↓	↓↓	↑
	Δchl1 Δrad8	-	-	↓↓	-	↑
	Δsrs2 Δrad8	-	↑	↓	-	↓↓
	Δsnf22 Δrad8	-	↑	-	-	↓

The level of sensitivity is scored by the fitness on the drug plates. No difference from the either of the single mutants is labeled as "-". The level of increased drug sensitivity is scored by number of "↓". The level of reduced drug sensitivity is scored by number of "↑". ND = not determined. $^{\$}$ one RNA helicase partially decreased the MMS sensitivity of rad8. SPBC3B8.12 = SPBC11C11.11c.
