# Supplementary material for: Essential Domains of Schizosaccharomyces pombe Rad8 Required for DNA Damage Response
Source: G3 (Bethesda). 2014 May 28;4(8):1373–84. doi: 10.1534/g3.114.011346 (PMC4132169; doi:10.1534/g3.114.011346)
Supplement: Supporting Information [file supp_g3.114.011346_FigureS2.pdf]

Fig.S2

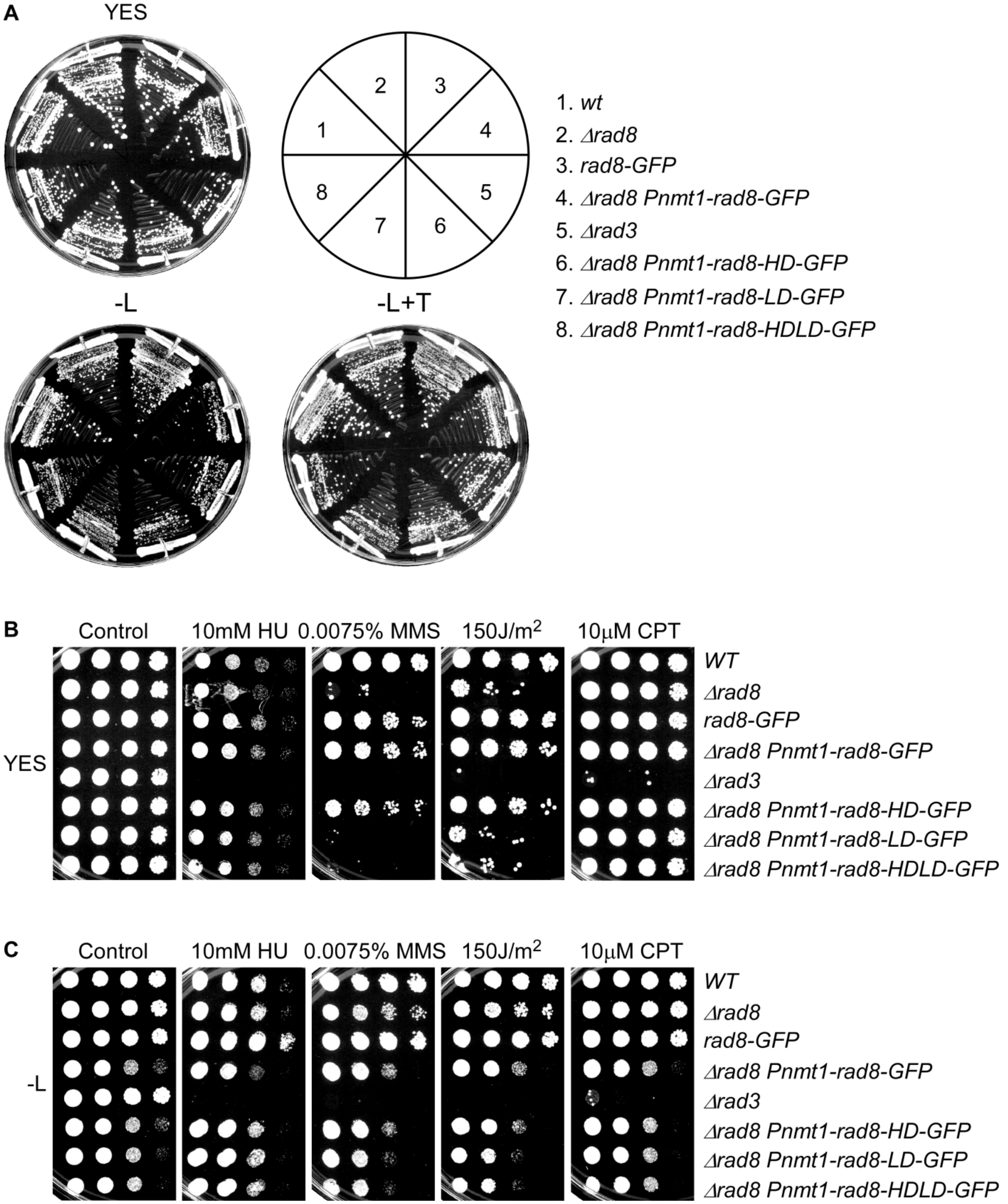

Figure S2 Overproduction of *rad8* in *Δrad8* has minor defects. (A) Overproduction (EMM-LEU plate) of *rad8-GFP* causes slightly growth defect. Strains were streaked out on different medium according to the schematics. (B) *rad8-GFP* fully complements Δ*rad8* and cells mount the same response to damaging drugs in the presence of thiamine (YES). Strains were grown overnight at 32ºC, 1:5 serially diluted and spotted to plain YES rich medium (Control) and YES with indicated drugs. (C) Overproduction of *rad8-GFP* and mutants is slightly toxic to cells. Strains were grown overnight at 32ºC, washed twice with EMM-LEU medium, 1:5 serially diluted in EMM-LEU and spotted to EMM-LEU medium (Control) and EMM-LEU with indicated drugs. Plates were incubated at 32ºC for 3 days if not indicated.
