# Supplementary material for: Essential Domains of Schizosaccharomyces pombe Rad8 Required for DNA Damage Response
Source: G3 (Bethesda). 2014 May 28;4(8):1373–84. doi: 10.1534/g3.114.011346 (PMC4132169; doi:10.1534/g3.114.011346)
Supplement: Supporting Information [file supp_g3.114.011346_FigureS3.pdf]

Fig.S3

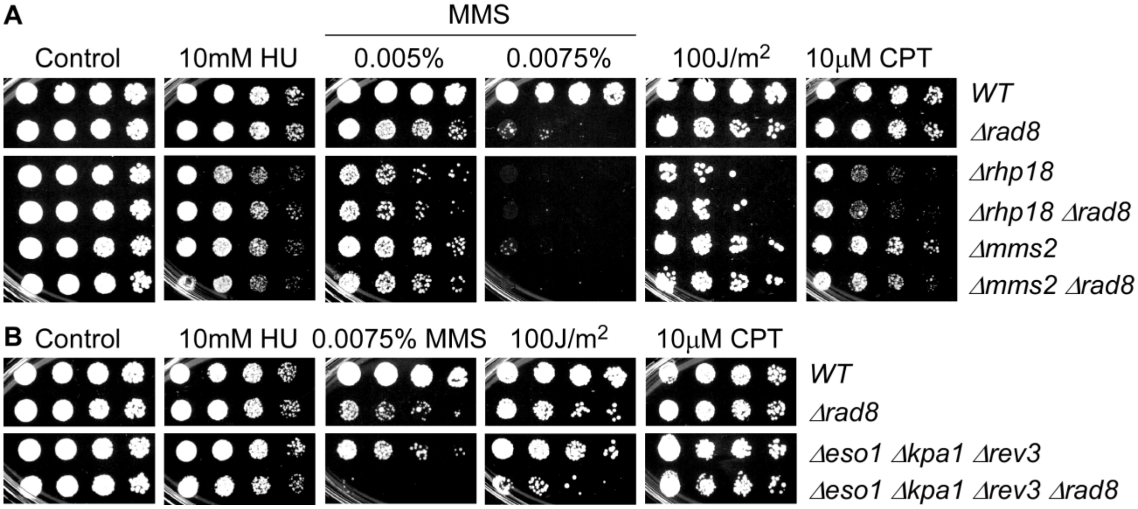

Figure S3 Rad8 functions in the PRR pathway. (A) *rad8*Δ is epistatic with *rhp18*Δ and *mms2*Δ. Strains were grown overnight at 32ºC, 1:5 serially diluted and spotted to plain YES rich medium (Control) and YES with indicated drugs. (B) *rad8*Δ is not epistatic with TLS mutants. Strains were grown overnight at 32ºC, 1:5 serially diluted and spotted to plain YES rich medium (Control) and YES with indicated drugs. Plates were incubated at 32ºC for 3 days.
