# Supplementary material for: Essential Domains of Schizosaccharomyces pombe Rad8 Required for DNA Damage Response
Source: G3 (Bethesda). 2014 May 28;4(8):1373–84. doi: 10.1534/g3.114.011346 (PMC4132169; doi:10.1534/g3.114.011346)
Supplement: Supporting Information [file supp_g3.114.011346_FigureS5.pdf]

Fig.S5

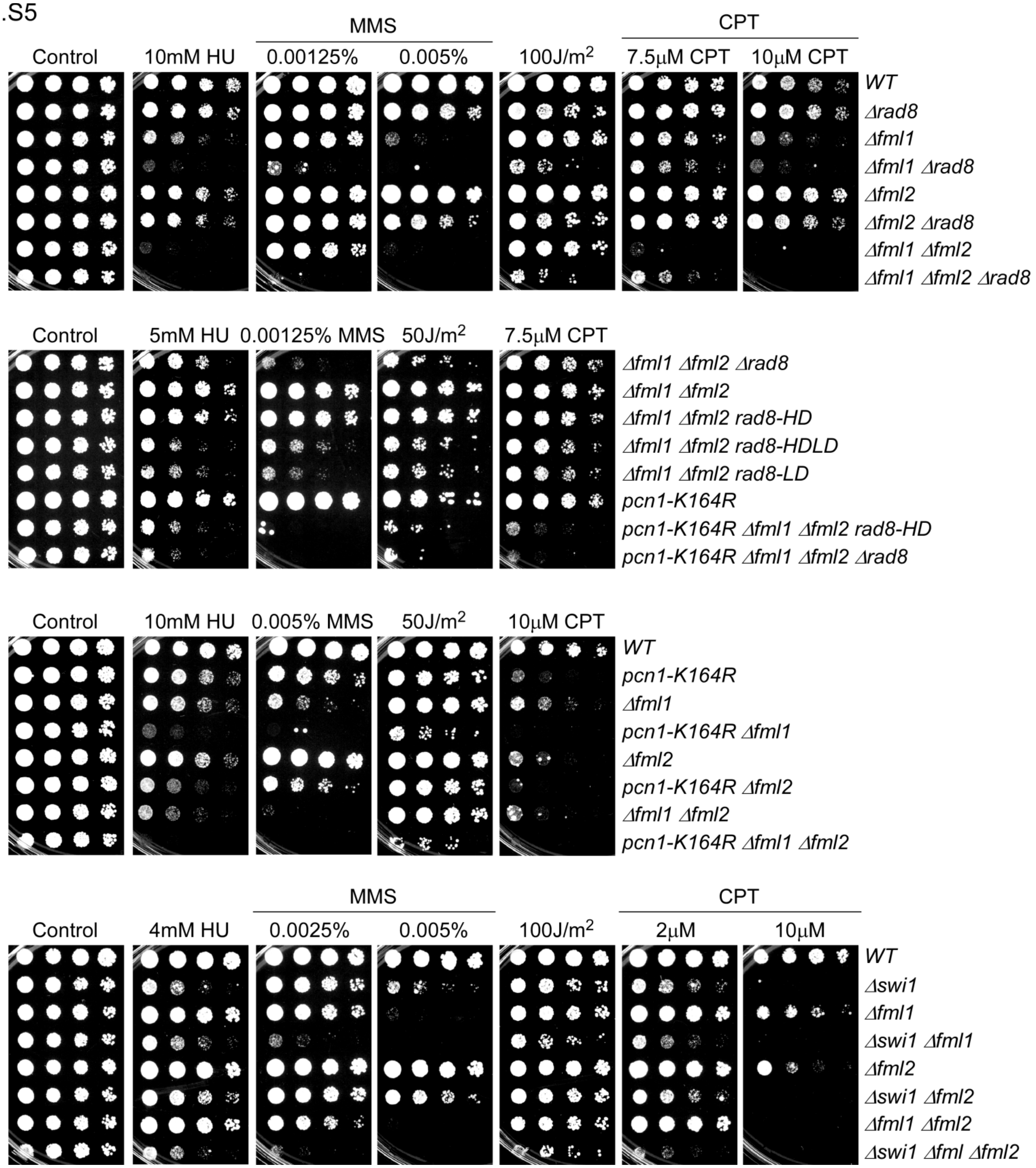

Figure S5 Fml1 and Rad8 ligase domain are functionally redundant. Strains were grown overnight at 32ºC, 1:5 serially diluted and spotted to plain YES rich medium (Control) and YES with indicated drugs. Plates were incubated at 32ºC for 3 days.
